# Loss of ACSL1 fuels ferroptosis resistance in clear cell renal carcinoma

**DOI:** 10.1080/15384047.2025.2567815

**Published:** 2025-10-06

**Authors:** Shangguo Wang, Yuxiong Wang, Bin Liu, Dan Zhang, Zehua Zhang, Hongxia Yang, Guangtao Li, Xiaodong Zhao, Jiaxin Liu, Qianhui Li, Yifan Song, Yanghe Zhang, Yishu Wang, Honglan Zhou

**Affiliations:** a Department of Urology II, The First Hospital of Jilin University, Changchun, Jilin, China; b Key Laboratory of Pathobiology, Ministry of Education, Jilin University, Changchun, Jilin, China

**Keywords:** ccRCC, ACSL1, ferroptosis, ROS, p53

## Abstract

**Background:**

Clear cell renal cell carcinoma (ccRCC), the most common kidney cancer subtype, is marked by lipid metabolism reprogramming and therapy resistance. Ferroptosis—an iron-dependent, lipid peroxidation-driven cell death—has gained attention as a therapeutic strategy. This study investigates the role of ACSL1, a key lipid metabolism enzyme, in ccRCC.

**Methods:**

Using TCGA/GEO datasets, qPCR, immunohistochemistry, and immunofluorescence, ACSL1 expression and clinical significance were analyzed. Functional assays with ACSL1-overexpressing ccRCC cells and a xenograft mouse model evaluated its impact on tumor behavior. Transcriptomics and lipidomics, alongside ROS, ferroptosis, and p53 inhibitors, were applied to uncover mechanisms.

**Results:**

ACSL1 is markedly downregulated in ccRCC and predicts poor prognosis. Overexpression suppressed proliferation and migration, induced cell death, and slowed tumor growth. Mechanistically, ACSL1 elevated ROS, activated p53, downregulated SLC7A11/GPX4, and triggered ferroptosis. Blocking ROS or p53 reversed these effects, confirming a ROS-p53-SLC7A11/GPX4 feedback loop.

**Conclusion:**

ACSL1 functions as a tumor suppressor in ccRCC by inducing ferroptosis via the ROS-p53-SLC7A11/GPX4 axis. It holds promise as a prognostic biomarker and therapeutic target in ccRCC.

## Introduction

Clear cell renal cell carcinoma (ccRCC) accounts for 70%−80% of renal malignancies. The 5-y survival rate for patients with metastatic ccRCC remains below 10%,^1^ a clinical conundrum attributed to its aggressive metastatic potential and resistance to conventional therapies. Owing to the vascular nature of RCC, VEGF-targeting tyrosine kinase inhibitors (e.g., sunitinib, pazopanib) and mTOR inhibitors (e.g., everolimus) are approved for the treatment of metastatic RCC, though responses remain limited (8–9 months PFS in first-line) with no predictive biomarkers available. Sunitinib remains the first-line standard.^2–4^ Cytokines (interferon-α/IL-2) were early RCC immunotherapies but showed limited efficacy and high toxicity. The current focus has shifted to checkpoint inhibitors (e.g., nivolumab, pembrolizumab) that target PD-1/PD-L1 or CTLA-4, which enhance T-cell activity. While nivolumab improved survival vs. everolimus in pretreated patients (CheckMate 025), response rates remained low (25%), with no reliable biomarkers.^5^^,^^6^ Combination therapies targeting multiple pathways (e.g., VEGF + mTOR) show superior efficacy over single agents in RCC but often increase toxicity. While some combinations, such as bevacizumab + everolimus are tolerable, optimal polypharmacy requires precise targeting of primary (VEGF) and secondary pathways (e.g., mTOR, MET).^6^^,^^7^ Emerging strategies combining checkpoint inhibitors with targeted therapies may overcome resistance by addressing distinct escape mechanisms, though toxicity and patient-specific sequencing remain key challenges. This dismal prognosis is correlated with intratumoral heterogeneity, metabolic reprogramming, and adaptive drug resistance mechanisms. Notably, ccRCC exhibits profound dysregulation of lipid metabolism. These metabolic alterations not only fuel tumor progression but also establish targetable vulnerabilities for therapeutic intervention.

Ferroptosis, an iron-dependent form of programmed cell death driven by lipid peroxidation, has emerged as a promising therapeutic target for treatment-resistant cancers.^8^ Drug resistance (intrinsic/acquired) remains a major obstacle in cancer therapy, but targeting ferroptosis has emerged as a promising strategy to overcome resistance. Ferroptosis inducers reverse resistance to various drugs (e.g., lapatinib, sorafenib),^9^ while xCT/GPX4 inhibition enhances sensitivity to chemo/radiotherapy by disrupting redox balance.^10^^,^^11^ Notably, ferroptosis induction effectively eliminates therapy-resistant high-mesenchymal cancer cells by counteracting their lipid peroxidation defense mechanisms.^12^ Significantly, ccRCC cells demonstrate increased susceptibility to ferroptosis inducers across multiple tumor models.^13^ These findings not only implicate ferroptosis in ccRCC pathogenesis but also suggest its targeted induction of ferroptosis may constitute a novel anticancer strategy. Nevertheless, the genetic determinants governing ferroptotic sensitivity in ccRCC remain incompletely characterized, with particular knowledge gaps persisting regarding upstream regulators that orchestrate lipid metabolism–ferroptosis crosstalk and their dynamic feedback mechanisms.

Tumor cells have evolved multiple defense mechanisms to alleviate lipid peroxidation stress and suppress ferroptosis. These encompass the SLC7A11/GPX4 axis, FSP1/CoQ system, the mitochondrial DHODH/CoQ pathway, the GCH1/BH4 cascade, and vitamin K-mediated protection.^14–16^ The SLC7A11/GPX4 axis, the first identified and most extensively studied anti-ferroptotic pathway, operates through SLC7A11-mediated cystine uptake for glutathione (GSH) biosynthesis, with its downstream effector GPX4 utilizing GSH as a cofactor to catalytically reduce lipid peroxides into non-toxic lipid alcohols, thereby preserving plasma membrane integrity.^13^^,^^17^^,^^18^ Crucially, GPX4 knockdown elevates lipid peroxidation, exacerbates ferroptosis, and effectively inhibits tumor growth.^19^ Intriguingly, the tumor suppressor TP53 exerts context-dependent dual regulation on ferroptosis: while p53 promotes ferroptosis by repressing SLC7A11 expression or upregulating SAT1 (spermidine/spermine N1-acetyltransferase 1) and GLS2 (glutaminase 2), which conversely suppresses ferroptosis through direct inhibition of DPP4 (dipeptidyl peptidase 4) activity or induction of CDKN1A/p21 (cyclin-dependent kinase inhibitor 1A) expression.^20^

Acyl-CoA synthetase long-chain family member 1 (ACSL1), a pivotal enzyme catalyzing long-chain fatty acid (LCFA) activation, exhibits context-dependent roles in cancer progression through dual regulation of lipid biosynthesis and peroxidation. While ACSL1 demonstrates upregulated expression in most malignancies, including hepatocellular carcinoma,^21^ colorectal cancer,^22^ and ovarian cancer,^23^ where it drives lipid metabolic reprogramming and protein epigenetic modifications to fuel tumor bioenergetics and biosynthesis, its expression is paradoxically downregulated in specific cancers, such as clear cell renal carcinoma^24^ and lung squamous cell carcinoma.^25^ Mechanistically, ACSL1 is involved in bidirectional ferroptosis regulation: in breast cancer models, ACSL1 facilitates the incorporation of conjugated linoleates (e.g., αESA) into neutral lipids (e.g., triglycerides), generating oxidation-prone lipid species that accumulate lipid peroxidation products and trigger ferroptosis.^26^ This pro-ferroptotic effect is recapitulated in chronic myeloid leukemia, where ACSL1 overexpression enhances lipid peroxidation.^27^ Conversely, in other tumor types, ACSL1 promotes myristoylation and membrane localization of ferroptosis suppressor protein 1 (FSP1), thereby augmenting fatty acid oxidation capacity, reducing lipid peroxidation burden, and ultimately conferring ferroptosis resistance.^28^

Bioinformatic analyses have revealed significant enrichment of fatty acid metabolism pathway genes and ferroptosis-related genes in the ACSL1-high subgroup of ccRCC.^29^ However, no studies have definitively established whether altered ACSL1 expression levels (either upregulation or downregulation) in ccRCC directly correlate with ferroptosis-mediated cell death enhancement and proliferation suppression. Our preliminary data demonstrate marked downregulation of ACSL1 in ccRCC tissues, which shows significant associations with advanced pathological grade, metastasis, and poor prognosis. While this observation aligns with the recognized metabolic vulnerability of ccRCC to lipid peroxidation, the underlying molecular mechanisms require rigorous validation. In this study, we hypothesize that ACSL1 modulates ferroptosis sensitivity in clear cell renal cell carcinoma (ccRCC) by selectively biosynthesizing pro-ferroptotic phosphatidylethanolamines (PEs), particularly those esterified with arachidonoyl (C20:4) and adrenoyl (C22:4) fatty acyl chains. Our findings demonstrate that ACSL1-driven lipid remodeling establishes a permissive environment for lethal lipid peroxidation, thereby positioning ACSL1 as a critical metabolic checkpoint in the susceptibility of ccRCC ferroptosis susceptibility. We further propose that ACSL1 acts as a tumor suppressor in ccRCC by reshaping lipid peroxidation homeostasis and activating an ROS-p53 positive feedback loop to promote ferroptosis. This study provides the first evidence underscoring ACSL1’s pivotal role in regulating ferroptosis in ccRCC, identifying it as a key metabolic node linking lipid remodeling to oxidative cell death. By elucidating these dynamic feedback regulatory networks, our work not only advances the mechanistic understanding of ferroptosis but also establishes a theoretical foundation for overcoming therapeutic resistance via co-targeting with ACSL1 activators and ferroptosis inducers. These insights hold significant translational potential for improving clinical outcomes in ccRCC.

### Methods

#### Source of human tissue samples

Human tumor tissues and paired adjacent non-cancerous tissues were obtained from patients undergoing routine surgical procedures in the Department of Urology, the Second Division of the First Hospital of Jilin University. These specimens, which were collected as part of standard clinical care rather than being prospectively acquired for research purposes, were provided by the hospital's Department of Biobank, Division of Clinical Research. The study protocol was approved by the Institutional Ethics Committee of the First Hospital of Jilin University (Approval No. 2023-KS-238).

### Cell line and culture method

The renal carcinoma cell lines A498 and 786O used in this study were obtained from the National Infrastructure of Cell Line Resource (China). A498 cells were maintained in DMEM supplemented with 10% fetal bovine serum (FBS) and 1% penicillin-streptomycin (P/S), while 786O cells were cultured in RPMI-1640 medium containing 10% FBS and 1% P/S.

Stable ACSL1-overexpressing cell lines (A498-OE-ACSL1 and 786O-OE-ACSL1) with their corresponding negative controls (A498-OE-NC and 786O-OE-NC) were generated using lentiviral overexpression vectors. Puromycin selection was performed at concentrations of 2  μg/mL for 786 O and 4 μg/mL for A498 over two weeks to establish resistant monoclonal populations, followed by expansion through serial passaging.

### Subcutaneous tumorigenesis assay in mice

All animal procedures were approved by the Institutional Ethics Committee of the First Hospital of Jilin University (Approval No. 2023-0236) and performed in strict accordance with ARRIVE guidelines. Female BALB/c-nu nude mice (6−8 weeks old) were purchased from SPF Biotechnology Co., Ltd. (Beijing, China). The animals were maintained in individually ventilated cages under controlled conditions (22 ± 1°C, 12 h light/dark cycle) with ad libitum access to autoclaved food and water.

Ten BALB/c-nu mice were randomly allocated into two groups: the CTL group (*n* = 5), which included 786O cells transfected with the empty vector, and the OE-ACSL1 group (*n* = 5), which included 786O cells stably overexpressing ACSL1. A single-cell suspension containing 4 × 10⁶ cells in 200 μL of PBS was subcutaneously injected into the right flank of each mouse. Tumor dimensions were measured twice weekly using digital calipers starting from post-implantation day 10−14 when palpable tumors became detectable. The tumor volume was calculated as 0.52 × length (mm) × width² (mm). Humane endpoints were strictly enforced with immediate euthanasia by CO₂ asphyxiation when the tumor volume exceeded 1500 mm³ or when ulceration developed. After 6−7 weeks, all the mice were sacrificed, and the tumors were excised for gravimetric analysis and subsequent processing.

### Bioinformatics analysis

The mRNA count data and clinical records of ccRCC patients from the TCGA-KIRC cohort were retrieved through Sangerbox 3.0 platform (http://vip.sangerbox.com/home.html) to construct a raw mRNA expression matrix. The raw count data were normalized using the platform's standardization module. Systematic comparisons were performed to evaluate ACSL1 expression differences across three comparative groups: (1) tumor vs. normal tissues, (2) lymph node metastasis-positive vs. -negative subgroups, and (3) distinct histologic grade categories.

UALCAN database (https://ualcan.path.uab.edu/analysis.html) was employed to analyze differential expression of ACSL1 protein between tumor and normal tissues, as well as mRNA-level variations between ccA and ccB molecular subtypes in ccRCC.

Gene Expression Omnibus (GEO) datasets (GSE6344, GSE36895) were downloaded from https://www.ncbi.nlm.nih.gov/geo/ to validate ACSL1 expression discrepancies in tumor versus normal tissues.

The association between ACSL1 mRNA expression and overall survival in ccRCC patients was assessed using GEPIA database (http://gepia2.cancer-pku.cn/#survival), with Kaplan-Meier curves generated accordingly.

Additional validation was conducted through analysis of E-MTAB-1980 ccRCC expression dataset from ArrayExpress (https://www.ebi.ac.uk/arrayexpress/experiments/E-MTAB-1980/). GraphPad Prism 9 was utilized for Kaplan‒Meier curve plotting, receiver operating characteristic (ROC) curve construction, and area under the curve (AUC) calculation.

### Transcriptome sequencing analysis

The transcriptome sequencing analysis was performed by Novogene Co. Ltd. (Beijing, China). The experiment included control (A498-OE-NC) and ACSL1-overexpressing (A498-OE-ACSL1) groups, each comprising three biological replicates to ensure experimental reliability and reproducibility.

Differentially expressed genes (DEGs) were identified using the edgeR package with Benjamini‒Hochberg correction for multiple hypothesis testing. KEGG (Kyoto Encyclopedia of Genes and Genomes) pathway enrichment analysis of DEGs was conducted using clusterProfiler, with false discovery rate (FDR)-adjusted *p*-values < 0.05 was considered statistically significant.

### Untargeted lipidomics analysis

The untargeted lipidomics analysis was conducted by Novogene Co., Ltd. (Beijing, China). The experimental samples comprised control (A498-OE-NC) and ACSL1-overexpressing (A498-OE-ACSL1) groups, each containing six biological replicates. The samples were reconstituted in isopropanol and analyzed via liquid chromatography‒tandem mass spectrometry (LC‒MS‒MS). Chromatographic separation was performed using a specified column under optimized conditions, while mass spectrometry detection was performed in both positive and negative ionization modes.

 Raw data were processed using LipidSearch software for peak extraction, lipid identification/quantification, inter-sample peak alignment, background ion subtraction based on blank samples, and data normalization. Significant differential metabolites were identified through multivariate statistical analyses (principal component analysis, PCA; partial least squares-discriminant analysis, PLS-DA) and univariate analysis (Student’s *t*-test). Visualization was achieved via volcano plots, hierarchical clustering heatmaps, and correlation networks.

### Western blot analysis, immunohistochemistry, and immunofluorescence

Kidney tissue or cell lysates were prepared using RIPA buffer (Solarbio, China, R0020) supplemented with a 1% protease inhibitor mixture (Solarbio, China, P6730) and 1% phosphatase inhibitor (Solarbio, China, P1260). The tissues were homogenized using a homogenizer. The cell lysate was separated using centrifugation and equal amounts of protein were loaded onto the gel. Proteins were transferred onto the PVDF membrane (Merck, Germany, ISEQ00010) and probed with the antibodies detailed in Supplementary Table 1. The next day, the membranes were washed three times with Tris-buffered saline with Tween (TBST) and incubated with secondary antibodies (HRP-conjugated AffiniPure goat anti-rabbit IgG (Proteintech, China, SA00001-2) or HRP-conjugated AffiniPure goat anti-mouse IgG (Proteintech, China, SA00001-1)) for 1 h at room temperature. The samples were then developed with Meilunbio® fg supersensitive ECL luminescence reagent (Solarbio, China, ECL Western Blotting Substrate, PE0010a) after being washed three times with TBST. The ECL imaging equipment includes the Tanon 4600 (Shanghai, China) and the GeneGnome HR model No. 7000 (Synoptics Ltd., UK).

 Collected tissues were fixed in formalin for 1 week, and paraffin-embedded sections were prepared for subsequent histologic examination.

The initial steps for both immunohistochemical staining and immunofluorescence staining were identical. The paraffin-embedded sections were first dewaxed and hydrated. The antigens were retrieved in 10 mM sodium citrate solution at high temperature (95 °C) for 5 min and then cooled to room temperature.

For immunohistochemical staining, endogenous peroxidase activity was quenched using 3% H_2_O_2_ for 15 min. The sections were subsequently blocked with 0.2% fish skin gelatin for 30 min at room temperature. The samples were then incubated overnight at 4 °C with primary antibodies (details regarding specific antibody types and dilution concentrations are provided in Supplementary Table 1). The following day, after a 15-min incubation with secondary antibodies at room temperature, a streptavidin‒peroxidase solution was applied for an additional 15 min. Finally, staining was visualized using either 3,3'-diaminobenzidine (DAB, Solarbio, DA1016) or 3-amino-9-ethylcarbozole (AEC, Solarbio, A2010). Finally, hematoxylin staining, differentiation and bluing were used to label the nucleus. It is noteworthy that all the mentioned reagents were sourced from the SP kits (Broad Spectrum) provided by Solepol (SP0041).

For immunofluorescence staining, the sections were initially blocked with 5% normal goat serum for 30 min at room temperature. The sections were subsequently incubated overnight at 4 °C with primary antibodies (specific antibody types and dilution concentrations are provided in Supplementary Table 1). On the following day, the sections were washed with PBS and incubated with fluorescent secondary antibodies (see Supplementary Table 1 for details). Additionally, the nuclei were stained with DAPI, and the proximal tubules were stained with fluorescein lotus tetragonolobus lectin (LTL, 1:200, FL1321, Vector Laboratories) for 60 min.

### Real-time quantitative polymerase chain reaction analysis

We extracted total RNA using the Total RNA Extractor kit (Solarbio, China, R1200). Subsequently, 1 μg of total RNA was reverse-transcribed using the cDNA Synthesis SuperMix Kit (YEASEN, China, 11137ES60). Real-time quantitative polymerase chain reaction (RT-qPCR) analysis was conducted using the Hieff® System (YEASEN, China, 11203ES08) with SYBR Green Master Mix (High Rox Plus), following 3-step standard cycling conditions and employing the sequence-specific primers listed in Supplementary Table 2. Melting curve analysis was performed to confirm the amplification of a single product. For quantitative analysis, all samples were normalized to GAPDH gene expression using the ΔΔCT method.

### Pharmacological compounds and test kits

The information of various test kits and compounds used in this study is listed in Supplementary Table 3.

### Statistical analysis

The data are presented as mean ± standard deviation (SD). Differences between two groups were analyzed using an unpaired or paired Student’s *t*-test, as appropriate, after confirming that the data met the assumption of normality. The fluorescence intensity was semi-quantified using ImageJ software. Statistical analyses and graph generation were performed with GraphPad Prism 9.0. All in vitro experiments included three independent biological replicates. The in vivo studies included five animals per group. Statistical significance was defined as *p *< 0.05.

## Results

### ACSL1 suppression associates with tumor aggressiveness and prognosis in ccRCC

**Figure 1.**
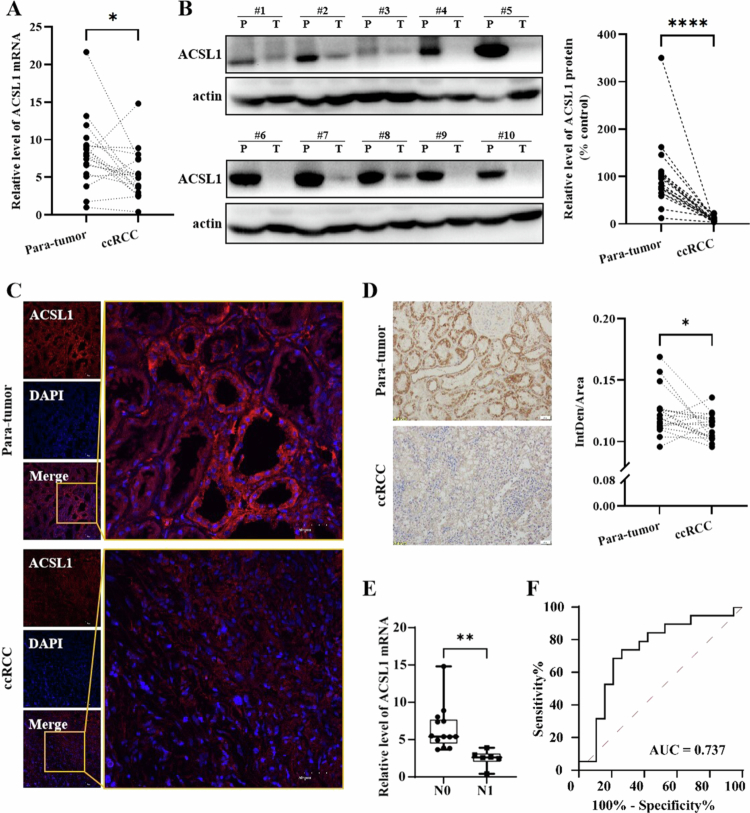
Association between ACSL1 suppression and tumor aggressiveness/prognostic significance in ccRCC. (A) Paired comparison of ACSL1 mRNA levels in clinical ccRCC specimens and adjacent normal renal tissues (*n* = 19). (B) Representative Western blot images with quantitative analysis showing ACSL1 protein expression in matched samples. (C) Immunofluorescence staining of ACSL1 in paired specimens. Scale bars = 100 μm (overview), 50  μm (magnified view). (D) IHC profiles and corresponding quantification of ACSL1 expression in matched tissues. Scale bar = 20 μm. (E) Comparative analysis of ACSL1 mRNA levels between lymph node metastasis-positive (N1) and -negative (N0) ccRCC cases. (F) ROC curve demonstrating the diagnostic potential of ACSL1 expression for discriminating tumor tissues from normal counterparts. *P < 0.05, **P < 0.01, ***P < 0.001, ****P < 0.0001.

To investigate the role of ACSL1 in ccRCC, we first analyzed its expression in 19 paired tumor and adjacent normal tissues. qPCR revealed significantly lower ACSL1 mRNA levels in tumor tissues (Figure 1A), a finding validated in the TCGA-KIRC cohort (506 tumors vs. 72 normal controls; Supplementary Figure 1A) and two independent GEO datasets (GSE36895/GSE6344; Supplementary Figure 1B). Furthermore, we found that the expression of ACSL1 in normal renal tubular cells was greater than that in 786O cells and A498 cells (Supplementary Figure 1G and 1H).

 Western blot analysis demonstrated reduced ACSL1 protein expression in tumor tissues (Figure 1B). Immunofluorescence showed predominant cytoplasmic localization with diminished intensity in tumors (Figure 1C). IHC confirmed stronger staining in normal tissues (Figure 1D), corroborated by CPTAC proteomic data (Supplementary Figure 1C).

Clinical correlation analysis revealed significant associations between low ACSL1 expression and malignant features: a negative correlation with lymph node metastasis (*n* = 19, Figure 1E), lower expression in N1 vs N0 patients (TCGA, Supplementary Figure 1D), and a progressive decrease with higher Fuhrman grade (Supplementary Figure 1E). Notably, aggressive ccB subtypes exhibited lower ACSL1 than ccA subtypes (Supplementary Figure 1F).

Survival analysis using TCGA data (GEPIA2) revealed shorter OS and DFS in patients with low ACSL1 expression (Supplementary Figure 2A), which was validated in E-MTAB-1980 cohort (Supplementary Figure 2B). Diagnostic ROC analysis yielded AUCs of 0.737 (current cohort, Figure 1F), 0.816 (TCGA validation, Supplementary Figure 2C), and 0.820 (GEO validation, Supplementary Figure 2D) for tumor-normal discrimination.

Our findings establish ACSL1 downregulation as a hallmark of ccRCC progression, correlating with advanced tumor grade, metastatic potential, and poor prognosis, while highlighting its diagnostic and prognostic utility.

#### ACSL1 impairs ccRCC tumorigenesis through dual suppression of proliferation and migration

Building on the established prognostic value of ACSL1 in ccRCC, we investigated its functional role using stable overexpression models in A498 and 786O cells (OE vs CTL; Supplementary Figure 3A, B).

**Figure 2.**
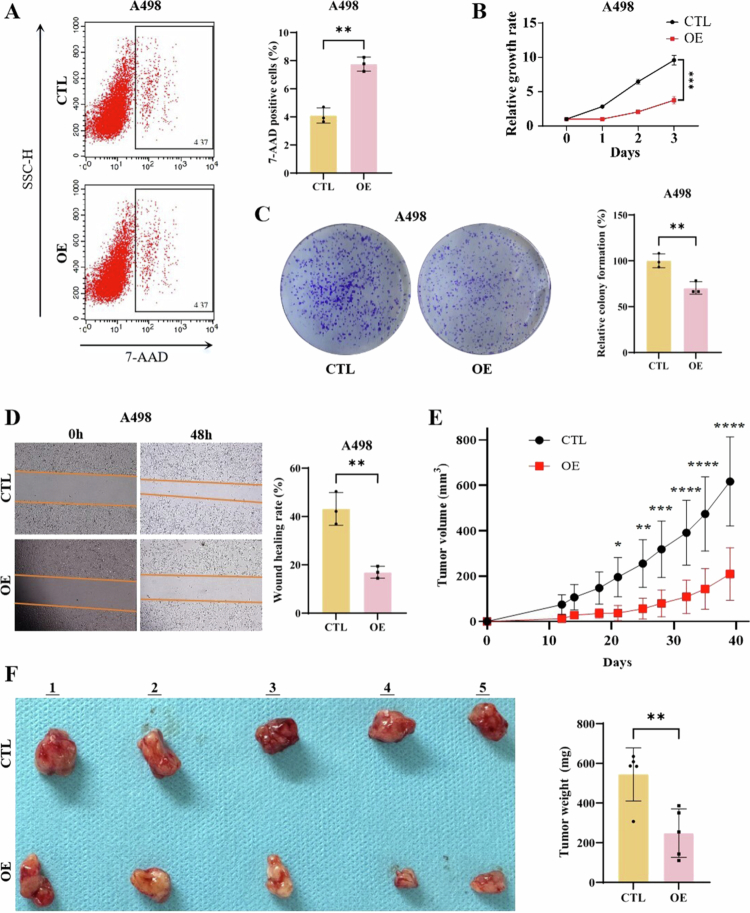
ACSL1 suppresses tumor progression in ccRCC through inhibiting proliferation and migration. (A) Flow cytometric analysis of 7-AAD-positive A498 cells (left) and corresponding quantitative analysis in A498 cells (right). (B) CCK-8 cell proliferation assay in A498 cells. (C) Representative colony formation assay images (left) and quantification (right) in A498 cells. (D) Scratch wound healing assay images (left) and migration rate quantification (right) at the 48-h timepoint in A498 cells. (E) Tumor growth kinetics in A498 cell-derived subcutaneous xenograft models over 39 d (*n* = 5 per group). (F) Gross morphology of excised tumors (left) and tumor weight quantification (right) at the endpoint (*n* = 5 per group). *P < 0.05, **P < 0.01, ***P < 0.001, ****P < 0.0001.

In vitro analyses demonstrated that ACSL1 overexpression: (i) increased 7-AAD^+^ cell population indicating impaired viability (Figure 2A; Supplementary Figure 3C); (ii) reduced proliferation rate at 72 h by CCK-8 assay (Figure 2B; Supplementary Figure 3D); (iii) decreased colony formation capacity (Figure 2C; Supplementary Figure 3E); and (iv) suppressed migration capacity at 48 h (Figure 2D; Supplementary Figure 3F). In vivo, subcutaneous xenograft models (*n* = 5/group) revealed significantly slower tumor growth in OE group (Figure 2E), with final tumor weight reduced by 54.3% (544.4 vs. 248.6 mg, *P *< 0.01; Figure 2F). Moreover, we constructed cells with ACSL1 knockout (Supplementary Figure 3G and 3H) and conducted clone formation (Supplementary Figure 3I and 3J) and scratch assays (Supplementary Figure 3K and 3L) to rule out the non-biological effects caused by overexpression of the gene.

These results mechanistically establish ACSL1 as a tumor suppressor in ccRCC through coordinated inhibition of proliferation and migration pathways, highlighting its therapeutic potential.

#### ACSL1 triggers ferroptotic cell death through lipid peroxidation remodeling in ccRCC

**Figure 3.**
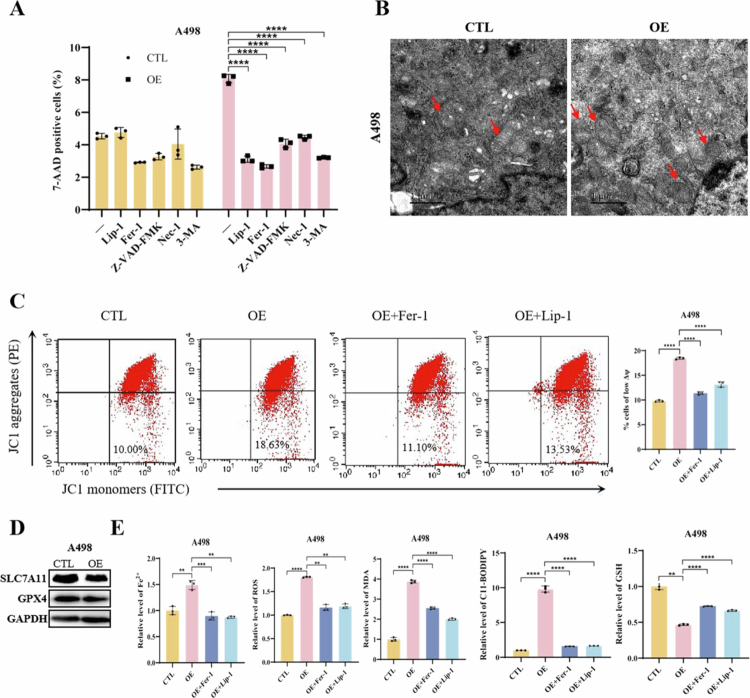
ACSL1 promotes ferroptosis in ccRCC cells. (A) Flow cytometric analysis of 7-AAD-positive cells in A498 cells treated with ferroptosis inhibitors (Lip-1, 500 nM; Fer-1, 10 μM), an apoptosis inhibitor (Z-VAD-FMK, 10 μM), a necroptosis inhibitor (Nec-1, 10  μM), and an autophagy inhibitor (3-MA, 2 mM). (B) Transmission electron microscopy images of the A498 cellular ultrastructure. The red arrowheads indicate mitochondria (11,500× magnification). Scale bar = 1 μm. (C) JC-1 fluorescence intensity analysis (left) and quantitative assessment of the mitochondrial membrane potential (right) in treated A498 cells. (D) Western blot analysis of the ferroptosis-related proteins SLC7A11 and GPX4 in ACSL1-overexpressing A498 cells. (E) Biochemical profiles of iron metabolism and oxidative stress markers: Fe²⁺ levels; ROS, MDA, C11-BODIPY oxidation; and GSH content in the experimental groups. *P < 0.05, **P < 0.01, ***P < 0.001, ****P < 0.0001.

Our initial observation that ACSL1 overexpression significantly increased ccRCC cell mortality (Figure 2A, Supplementary Figure 3C) prompted systematic investigation of its cell death mechanism. Pharmacological screening revealed that ferroptosis inhibitors (Lip-1 and Fer-1) effectively rescued ACSL1-induced cell death (Figure 3A, Supplementary Figure 4A), whereas apoptosis (Z-VAD-FMK), necroptosis (Nec-1), and autophagy inhibitors (3-MA) showed limited efficacy, establishing ferroptosis as the predominant mechanism.

Transmission electron microscopy revealed classic ferroptotic features in ACSL1-overexpressing cells, including shrunken mitochondria with cristae disappearance (Figure 3B, Supplementary Figure 4B). JC-1 assays confirmed a significant reduction in the mitochondrial membrane potential upon ACSL1 overexpression, which was reversed by ferroptosis inhibitors (Figure 3C, Supplementary Figure 4C). These data collectively demonstrate ferroptosis as the primary cell death modality driven by ACSL1.

Mechanistically, ACSL1 overexpression substantially downregulated the core ferroptosis suppressors SLC7A11 and GPX4 (Figure 3D, Supplementary Figure 4D), inducing iron dysregulation characterized by elevated Fe²⁺, ROS, lipid peroxidation markers (MDA, C11-BODIPY), and concurrent GSH depletion – all rescued by Lip-1/Fer-1 treatment (Figure 3E, Supplementary Figure 4E).

**Figure 4.**
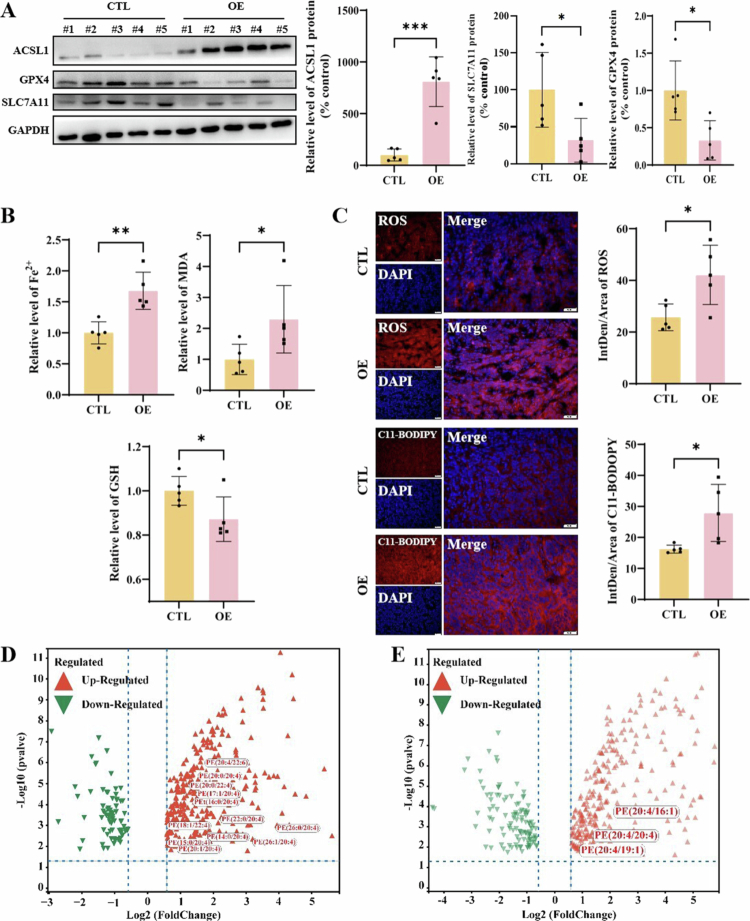
In vivo validation of ACSL1 to promote ferroptosis in ccRCC xenografts. (A) Detection and statistical analysis of the ferroptosis marker proteins SLC7A11 and GPX4 in xenografts overexpressing ACSL1. (B) Measurements of Fe²⁺, MDA, and GSH levels in xenograft tissues. (C) ROS and C11-BODIPY levels in frozen xenograft sections were assessed by fluorescent probes, with quantitative analysis. Scale bar = 50 μm. (D, E) Untargeted lipidomics analysis showing significant elevation of PE species containing arachidonic acid (C20:4) or adrenic acid (C22:4) in the OE group under negative-ion (D) and positive-ion (E) modes. *P < 0.05, **P < 0.01, ***P < 0.001, ****P < 0.0001.

In vivo validation showed suppressed SLC7A11/GPX4 expression (Figure 4A), Fe²⁺, MDA accumulation, GSH reduction (Figure 4B), and enhanced ROS, C11-BODIPY (Figure 4C) in ACSL1-overexpressing xenografts, recapitulating the in vitro phenotypes and reinforcing ACSL1's pro-ferroptotic role in ccRCC.

Recent lipidomic insights into ferroptosis execution, untargeted lipidomics of OE versus CTL A498 cells revealed minimal intra-group metabolic variation but marked inter-group differences (Supplementary Figure 5A, B). Strikingly, multiple phosphatidylethanolamines (PEs) containing arachidonic acid (C20:4) and adrenic acid (C22:4) were significantly enriched in OE cells (Figure 4D, E), with differential lipids mapping to arachidonic acid metabolism (Supplementary Figure 5C, D). These findings establish ACSL1-driven lipid remodeling through the accumulation of ferroptotic PEs, which directly links lipid homeostasis disruption to the execution of oxidative damage during ferroptosis.

#### ACSL1 orchestrates ferroptosis via p53-mediated repression of SLC7A11/GPX4 in ccRCC

**Figure 5.**
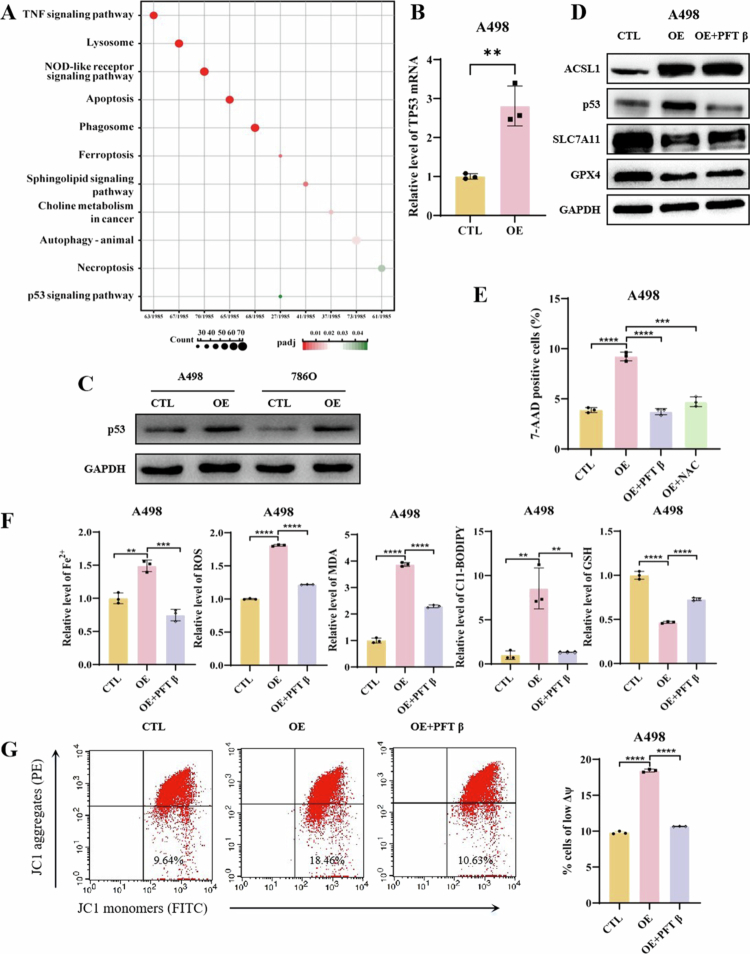
ACSL1 promotes ferroptosis in ccRCC through the p53-mediated SLC7A11/GPX4 axis. (A) Bubble plot of KEGG pathway enrichment analysis for upregulated genes from transcriptome sequencing. (B) TP53 mRNA expression levels in A498 cells upon ACSL1 overexpression. (C) Protein expression levels of p53 in A498 cells after ACSL1 overexpression. (D) SLC7A11 and GPX4 protein levels in A498 cells treated with the p53-specific inhibitor PFTβ (10 μM). (E) Flow cytometry quantification of 7-AAD-positive A498 cells after treatment with PFTβ and the ROS scavenger NAC (5 mM). (F) Measurements of Fe²⁺, ROS, MDA, C11-BODIPY, and GSH levels in A498 cells following PFTβ treatment. (G) JC-1 staining for mitochondrial membrane potential and corresponding quantification in PFTβ-treated A498 cells. *P < 0.05, **P < 0.01, ***P < 0.001, ****P < 0.0001.

To elucidate the molecular mechanism underlying ACSL1-regulated ferroptosis, we performed transcriptome sequencing of OE versus CTL A498 cells. Differential expression analysis revealed 1,809 significantly altered genes (762 upregulated, 1,047 downregulated). The upregulated genes were enriched in p53 signaling, ferroptosis, apoptosis, autophagy, and lipid metabolism pathways (Figure 5A, Supplementary Figure 6A, B), which is consistent with the observed multimodal cell death phenotypes. Notably, activation of the canonical tumor-suppressive p53 pathway suggested its potential involvement in regulating downstream ferroptosis effectors. The downregulated genes primarily participated in lipid and energy metabolism (Supplementary Figure 6C), validating lipidomic findings and indicating that ACSL1-driven metabolic reprogramming facilitates ferroptosis initiation.

The convergence of transcriptomic and lipidomic alterations prompted our hypothesis that ACSL1 disrupts metabolic homeostasis and oxidative phosphorylation to activate cell death pathways, with the p53 axis as a central regulator. Given the established roles of p53 in ferroptosis through SLC7A11/GPX4 suppression,^30^^,^^31^ we examined p53 expression in ACSL1-overexpressing cells. ACSL1 overexpression significantly upregulated *TP53* mRNA and p53 protein levels (Figure 5B, C, Supplementary Figure 7A). Pharmacological inhibition of p53 with pifithrin-*β* (PFTβ) restored SLC7A11/GPX4 protein expression (Figure 5D, Supplementary Figure 7B) and reversed ACSL1-induced cell death (reduced 7-AAD^+^ cells, Figure 5E, Supplementary Figure 7C). Ferroptosis marker analysis confirmed that PFTβ treatment attenuated Fe²⁺ levels, ROS, and lipid peroxidation products (MDA, C11-BODIPY), and while partially restoring the GSH content (Figure 5F, Supplementary Figure 7D) and rescuing the mitochondrial membrane potential (JC-1 aggregate recovery, Figure 5G, Supplementary Figure 7E). To explore the potential regulatory effects of p53 on SLC7A11 and GPX4 expression, we performed in silico analysis of their promoter regions using the JASPAR database (http://jaspar.elixir.no/). Bioinformatics prediction revealed putative p53-binding sites in both promoters (Supplementary Figure S8E and S8F).

Integrating multi-omics data, we propose a dual-mechanism model: (1) Transcriptional activation of p53 signaling suppresses SLC7A11/GPX4 expression; (2) metabolic remodeling creates a lipid peroxidation-prone microenvironment (Figure 4D, E). This synergy drives lipid radical accumulation beyond cellular repair capacity, culminating in irreversible ferroptosis execution.

#### ACSL1 amplifies ferroptotic signaling through a ROS-p53 feedforward circuit

**Figure 6.**
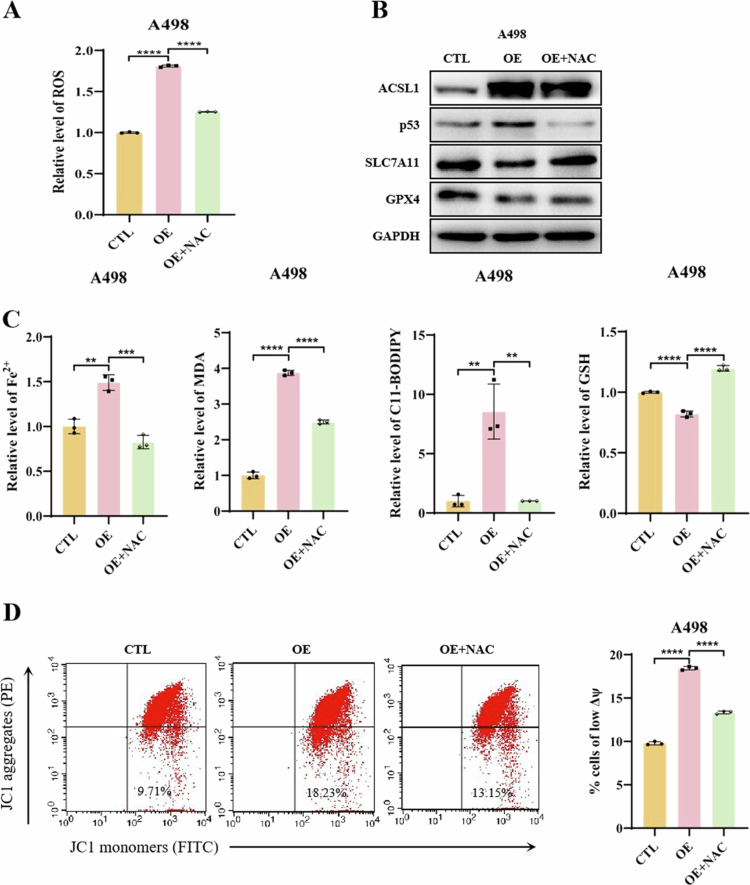
Attenuation of ACSL1-induced ferroptosis by reducing intracellular ROS. (A) Flow cytometry quantification of ROS levels in A498 cells treated with the ROS scavenger NAC (5 mM). (B) Representative Western blot images showing the protein expression of p53, SLC7A11, and GPX4 in NAC-treated A498 cells. (C) Measurements of Fe²⁺, MDA, C11-BODIPY, and GSH levels in A498 cells following NAC treatment. (D) JC-1 staining for determining the mitochondrial membrane potential in A498 cells treated with PFTβ, and the results were analyzed by flow cytometry. *P < 0.05, **P < 0.01, ***P < 0.001, ****P < 0.0001.

Given that ROS are key activators of the p53 signaling pathway^32^ and their progressive accumulation during ferroptosis, we hypothesized that ACSL1 might establish a ROS-p53-ferroptosis feedforward loop through ROS induction. In ACSL1-overexpressing ccRCC cells, treatment with the ROS scavenger *N*-acetylcysteine (NAC) markedly reduced intracellular ROS levels (Figure 6A, Supplementary Figure 8A). Notably, NAC administration not only reversed ACSL1-induced p53 upregulation but also restored SLC7A11 and GPX4 expression (Figure 6B, Supplementary Figure 8B), establishing ROS as the central mediator of p53 pathway activation by ACSL1.

To delineate the mechanistic role of ROS in ferroptosis induction, we analyzed key ferroptosis markers post-NAC treatment. NAC effectively attenuated ACSL1-driven accumulation of lipid peroxidation markers (MDA, C11-BODIPY) and intracellular Fe^2+^, while partially restoring GSH levels (Figure 6C, Supplementary Figure 8C). JC-1 staining revealed a significant improvement in the mitochondrial membrane potential following NAC treatment (Figure 6D, Supplementary Figure 8D).

Our findings collectively reveal a self-reinforcing regulatory circuit: ACSL1 activates p53 through ROS induction, which in turn exacerbates ferroptosis by repressing SLC7A11/GPX4 expression. The resulting oxidative stress from ferroptosis further amplifies the ROS-p53 signaling axis, establishing a positive feedback loop that enhances ferroptosis susceptibility in ccRCC, thereby constraining tumor progression.

#### Discussion

The ccRCC has been implicated as a metabolic disorder characterized by pervasive dysregulation of oxygen availability, nutrient homeostasis, iron metabolism, and energy production within tumor cells, suggesting that targeting pivotal metabolic nodes may drive therapeutic innovation.^33^ ACSL1 orchestrates LCFA activation through the catalysis of acyl-CoA formation, a biochemical prerequisite for generating metabolically active LCFA‒CoA derivatives. This rate-limiting reaction serves as the gateway for subsequent metabolic processes, including *β*-oxidation, phospholipid biosynthesis, and triglyceride synthesis.^34^^,^^35^ Emerging evidence from bioinformatics analyses reveals that ACSL1 overexpression is associated with favorable prognosis in specific ccRCC subtypes.^29^ However, current investigations predominantly rely on computational predictions, whereas compelling experimental validation of the mechanistic role of ACSL1 in ccRCC pathogenesis remains conspicuously absent.

This study reveals the first mechanistic evidence that ACSL1 exerts tumor-suppressive effects in ccRCC through remodeling lipid peroxidation and activating the ferroptosis pathway. Mechanistically, ACSL1 expression is markedly downregulated in ccRCC, and ACSL1 deficiency strongly correlates with advanced tumor stage, metastasis, and poor prognosis. This suppression is primarily attributable to CpG island hypermethylation, which reduces transcriptional activity and thereby diminishes ACSL1 expression.^29^ Furthermore, ACSL1 levels exhibit significant associations with VHL and PBRM1_p.R1010 mutations, while inversely correlated with HIF1-*α* mRNA abundance,^36^ suggesting that these genetic alterations may drive ccRCC tumorigenesis via ACSL1 inhibition. Notably, ACSL1 demonstrates a paradoxical role in cancer progression with marked tissue specificity. In breast and hepatocellular carcinomas, ACSL1 accelerates tumor proliferation and chemoresistance by enhancing lipid biosynthesis and energy metabolism.^35^^,^^37^ Conversely, in chronic myeloid leukemia, elevated ACSL1 triggers leukemia cell senescence and sensitizes cells to imatinib by suppressing SIRT1 to activate the p53/p21 axis.^38^ Similarly, ACSL1 inhibition exacerbates malignant phenotypes in lung squamous cell carcinoma.^22^^,^^25^ These findings collectively suggest that the functional duality of ACSL1 may be governed by tumor type-specific metabolic niches. In ccRCC, characteristic lipid metabolic disorders, particularly VHL deficiency-induced oxidative stress vulnerability, may redirect ACSL1's substrate preference from lipid storage toward peroxidation pathways through altered subcellular localization. The overexpression of ACSL1 significantly increased cell mortality while reducing the proliferation rate and impairing the scratch healing capacity in ccRCC cells. In xenograft models, ACSL1-overexpressing tumors exhibit slower growth kinetics and lower final tumor weight compared to controls.

Recent lipidomic advances have identified arachidonic acid (AA, C20:4)- and adrenic acid (AdA, C22:4)-containing PEs as critical mediators of ferroptosis.^39^^,^^40^ Our untargeted lipidomics revealed increases in PE (20:4) and PE (22:4) levels, with concomitant activation of AA metabolic pathways upon ACSL1 overexpression. This finding aligns with reported AA metabolic regulation by ACSL1 in lung adenocarcinoma.^41^ Transcriptomic profiling demonstrated ACSL1-induced gene expression remodeling. The upregulated genes showed significant enrichment in ferroptosis, lipid metabolism, and p53 signaling, whereas the downregulated genes were enriched in lipid biosynthesis and oxidative phosphorylation. These findings suggest that ACSL1 exerts tumor-suppressive effects by disrupting lipid homeostasis and increasing p53-mediated ferroptosis susceptibility in ccRCC.

We treated ACSL1-overexpressing cells with inhibitors targeting necrosis, apoptosis, autophagy, and ferroptosis. Ferroptosis inhibition exhibited the most pronounced effect on cell viability. Transmission electron microscopy revealed characteristic ferroptotic features, including shrunken mitochondrial cristae, reduced volume, and increased double-membrane density.^13^^,^^42^ ACSL1 overexpression significantly downregulated SLC7A11 and GPX4 protein expression. Lipid peroxidation assays showed ACSL1-overexpressing cells presented increased ROS levels, increased C11-BODIPY fluorescence, and elevated MDA content, accompanied by increased ferrous iron and reduced GSH levels. Notably, ACSL1 overexpression upregulated TP53 mRNA and p53 protein levels. Mechanistically, the p53 inhibitor PFTβ reversed the ACSL1-mediated suppression of SLC7A11 and GPX4, while significantly reducing cell death. Previous studies have shown that impaired mitochondrial quality control-induced ROS accumulation can promote p53 upregulation.^32^ Treatment with the ROS scavenger NAC significantly attenuated both the ROS and p53 protein levels in ACSL1-overexpressing ccRCC cells, while restoring SLC7A11 and GPX4 protein expression along with ferroptosis-related parameters. These findings indicate that p53 expression is regulated by ROS in ACSL1-overexpressing ccRCC cells. Notably, although p53 mutation rates remain relatively low in ccRCC, its functional integrity may still be compromised by non-genotoxic stressors such as reactive oxygen species (ROS). Unlike canonical ferroptosis regulators such as NRF2 and HSPB1, ACSL1, as an upstream lipid metabolism modulator, exhibits unique regulatory capacity over the ROS-p53-SLC7A11/GPX4 axis.

Our data therefore support a dynamic positive feedback loop model: ACSL1 upregulation triggers rapid intracellular ROS accumulation, which activates the p53 signaling pathway. Activated p53 subsequently suppresses SLC7A11 and GPX4 expression, compromising cellular antioxidant defenses and exacerbating lipid peroxidation, thereby amplifying ROS levels to establish a self-reinforcing positive feedback loop that perpetuates ferroptosis. This mechanism not only explains the characteristic "threshold effect" and irreversibility of ferroptosis in ccRCC, but also provides a novel paradigm for synergistically regulating tumor cell death through metabolic reprogramming and oxidative stress. This study makes conceptual advances by establishing the first direct regulatory link between the lipid metabolic enzyme ACSL1 and ferroptosis, bridging a critical knowledge gap in lipid reprogramming-cell death crosstalk while offering new molecular insights into ferroptosis irreversibility. Furthermore, ACSL1 expression levels may serve as predictive biomarkers for ferroptosis therapy response and prognosis in ccRCC patients.

 This study has two primary limitations. First, the association between ACSL1 and ferroptosis markers was derived from a limited clinical cohort, underscoring the need for validation in larger, independent populations. Second, while our data suggest a link, they remain correlative and do not establish a causative role for the observed phosphatidylethanolamines (PEs) as direct ferroptosis inducers. Future investigations should aim to elucidate the underlying mechanisms, such as through ACSL1-targeted strategies – including small-molecule agonists or genetic approaches – to determine whether ACSL1 modulation can increase the sensitivity of ccRCC to ferroptosis inducers such as erastin or RSL3.

In conclusion, this work elucidates the novel mechanism whereby ACSL1 amplifies ferroptosis signaling through ROS-p53-SLC7A11/GPX4 positive feedback to suppress ccRCC progression, providing a transformative conceptual framework and actionable targets for metabolic-oxidative stress combination therapies.

## Ethical approval statement

Human tumor tissues and paired adjacent non-cancerous tissues were obtained from routine surgical procedures in the Department of Urology, Second Division of the First Hospital of Jilin University. All specimens were collected as part of standard clinical care rather than being prospectively acquired for research purposes. The study protocol was approved by the Institutional Ethics Committee of the First Hospital of Jilin University (Approval No. 2023-KS-238). All animal procedures were approved by the Institutional Ethics Committee of the First Hospital of Jilin University (Approval No. 2023-0236) and performed in strict accordance with ARRIVE guidelines. All the above experiments were conducted in accordance with the Helsinki Declaration.

## Acknowledgments

We extend our sincere gratitude to the editors and reviewers of Cancer Biology & Therapy for their insightful comments and suggestions. We also acknowledge the Department of Biobank, Division of Clinical Research, for providing the human tissue samples. The graphical abstract was created with BioRender (https://biorender.com/).

## Author contributions

Author **Shangguo Wang**: Writing – Original Draft.

Author **Yuxiong Wang**: Conceptualization.

Author **Bin Liu**: Investigation.

Author **Zhang Dan**: Methodology

Author **Zehua Zhang**: Formal Analysis.

Author **Hongxia Yang**: Visualization.

Author **Guangtao Li**: Resources.

Author **Xiongdong Zhao**: Supervision

Author **Jiaxin Liu**: Investigation.

Author **Qianhui Li**: Investigation.

Author **Yifan Song**: Investigation.

Author **Yanghe Zhang**: Writing – Review & Editing, Validation.

Author **Yishu Wang**: Writing – Review & Editing, Validation.

Author **Honglan Zhou**: Writing – Review & Editing, Validation.

All authors discussed the results and approved the final manuscript.

## Supplementary Material

Supplementary material **Supplementary Figure 1. Bioinformatic analysis revealed significant downregulation of ACSL1 expression in ccRCC.** (A) Comparison of ACSL1 mRNA levels between ccRCC and adjacent normal kidney tissues in the TCGA dataset. (B) ACSL1 mRNA expression analysis in ccRCC versus normal kidney tissues from GSE36895 and GSE6344 datasets. (C) Differential ACSL1 protein expression between ccRCC and normal kidney tissues in the CPTAC database. (D) ACSL1 mRNA levels in lymph node metastatic (N1) versus non-metastatic (N0) ccRCC samples from the TCGA dataset. (E) Association between ACSL1 mRNA expression and pathological grades in the TCGA ccRCC cohort. (F) ACSL1 mRNA expression across molecular subtypes in the TCGA dataset, with the ccB subtype showing poorer prognosis. (G) Differential ACSL1 protein expression between HK2 and A498 cells. (H) Differential ACSL1 protein expression between HK2 and 786O cells. *P < 0.05, **P < 0.01, ***P < 0.001, ****P < 0.0001.

Supplementary material **Supplementary Figure 2. ACSL1 expression is correlated with prognosis in ccRCC patients.** (A) Kaplan‒Meier curves showing the OS and DFS of ccRCC patients stratified by ACSL1 expression levels in the TCGA dataset. (B) OS analysis based on ACSL1 expression in the ccRCC cohort from the E-MTAB-1980 dataset. (C) ROC curve analysis evaluating the diagnostic performance of ACSL1 expression in distinguishing ccRCC tumor tissues from normal kidney tissues in the TCGA dataset. (D) Diagnostic efficacy of ACSL1 expression assessed by receiver operating characteristic (ROC) curve analysis in GSE36895 and GSE6344 datasets.

Supplementary material **Supplementary Figure 3. ACSL1 suppresses tumor progression in ccRCC by inhibiting proliferation and migration.** (A) ACSL1 mRNA expression was validated via qPCR in A498 and 786O cell lines with ACSL1 overexpression and was normalized to that of GAPDH. (B) Western blot analysis of ACSL1 protein levels in ACSL1-overexpressing A498 and 786O cells. (C) Flow cytometry analysis (right) and quantification (left) of 7-AAD-positive cells in 786O cells. (D) Cell proliferation was assessed by a CCK-8 assay in 786O cells. (E) Representative images (left) and quantitative analysis (right) of colony formation in 786O cells. (F) Scratch wound healing assay images (left) and migration rate quantification (right) in 786O cells at 48 h. (G) The overexpression and knockdown of ACSL1 were verified by Western blotting in 786O cells. (H) The overexpression and knockdown of ACSL1 were verified by Western blotting in A498 cells. (I) Representative images (left) and quantitative analysis (right) of colony formation in 786O cells. (J) Representative images (left) and quantitative analysis (right) of colony formation in A498 cells. (K) Scratch wound healing assay images (left) and migration rate quantification (right) in 786O cells at 48 h. (L) Scratch wound healing assay images (left) and migration rate quantification (right) in A498 cells at 48 h. *P < 0.05, **P < 0.01, ***P < 0.001, ****P < 0.0001.

Supplementary material **Supplementary Figure 4. Role of ACSL1 in promoting ferroptosis in ccRCC cells**. (A) Flow cytometric analysis of 7-AAD-positive cell proportions in 786O cells treated with ferroptosis inhibitors (500 nM Lip-1 or 10 μM Fer-1), an apoptosis inhibitor (10 μM Z-VAD-FMK), a necroptosis inhibitor (10 μM Nec-1), or an autophagy inhibitor (2 mM 3-MA). (B) Representative transmission electron microscopy images of 786O cells (magnification = 11,500×; scale bar = 1 μm). Red arrowheads indicate mitochondria. (C) Quantitative analysis of JC-1 fluorescence intensity in 786O cells across treatment groups by flow cytometry. (D) Western blot analysis of ferroptosis-related proteins (SLC7A11 and GPX4) in ACSL1-overexpressing 786O cells. (E) Measurements of Fe²⁺, ROS, MDA, C11-BODIPY, and GSH levels in 786O cells under the indicated conditions. *P < 0.05, **P < 0.01, ***P < 0.001, ****P < 0.0001.

Supplementary material **Supplementary Figure 5. Untargeted lipidomics analysis revealed ACSL1-induced enhancement of ferroptosis-related lipid metabolism.** (A, B) Principal component analysis (PCA) plots of untargeted lipidomics data in negative ion mode (A) and positive ion mode (B) (*n *= 6 per group). (C, D) Pathway enrichment analysis of differentially abundant lipids between the ACSL1-overexpressing (OE) and control (CTL) groups in negative ion mode (C) and positive ion mode (D).

Supplementary material **Supplementary Figure 6. Transcriptomics analysis suggested that ACSL1 exerts tumor-suppressive effects via the p53 pathway.** (A) Volcano plot and (B) heatmap of differentially expressed genes identified by transcriptomic sequencing (*n *= 3 per group). (C) Bubble plot of KEGG pathway enrichment analysis for downregulated genes.

Supplementary material **Supplementary Figure 7. ACSL1 exerts its tumor-suppressive role by activating p53.** (A) TP53 mRNA expression levels in 786O cells after ACSL1 overexpression. (B) Western blot analysis of SLC7A11 and GPX4 protein levels in 786O cells treated with the p53-specific inhibitor PFTβ (10 μM). (C) Flow cytometry quantification of 7-AAD-positive cells in 786O cells treated with PFTβ and the ROS scavenger NAC (5 mM). (D) Measurements of intracellular Fe²⁺, ROS, MDA, C11-BODIPY, and GSH levels in PFTβ-treated 786O cells. (E) Flow cytometry analysis of JC-1 staining (mitochondrial membrane potential) and corresponding statistical results in PFTβ-treated 786O cells. *P < 0.05, **P < 0.01, ***P < 0.001, ****P < 0.0001.

Supplementary material **Supplementary Figure 8. Attenuation of ACSL1-induced ferroptosis by reducing intracellular ROS.** (A) Flow cytometry quantification of ROS levels in 786O cells treated with the ROS scavenger NAC (5 mM). (B) Representative Western blot images showing the protein expression levels of p53, SLC7A11, and GPX4 in NAC-treated 786O cells. (C) Measurements of intracellular Fe²⁺, MDA, C11-BODIPY, and GSH levels in NAC-treated 786O cells. (D) Flow cytometry analysis of JC-1 staining (mitochondrial membrane potential) in PFTβ-treated 786O cells. (E) Bioinformatic analysis predicted potential p53-binding sites within the SLC7A11 promoter region. (F) Bioinformatic analysis predicted potential p53-binding sites within the GPX4 promoter region. *P < 0.05, **P < 0.01, ***P < 0.001, ****P < 0.0001.

Supplementary material Supplementary materials CLEAN COPY.

## Data Availability

The data that supports the findings of this study are available in the supplementary material of this article.

## Abbreviations

ccRCC: Clear cell renal cell carcinoma
GSH: glutathione
ACSL1: Acyl-CoA synthetase long-chain family member 1
ROC: receiver operating characteristic
AUC: area under the curve
DEGs: Differentially expressed genes
Pes: phosphatidylethanolamines
PFTβ: pifithrin-β
NAC: *N*-acetylcysteine
LCFA: long-chain fatty acid.
