# Supplementary figures and images for: Loss of ACSL1 fuels ferroptosis resistance in clear cell renal carcinoma
Source: Cancer Biol Ther. 2025 Oct 6;26(1):2567815. doi: 10.1080/15384047.2025.2567815 (PMC12505508; doi:10.1080/15384047.2025.2567815)

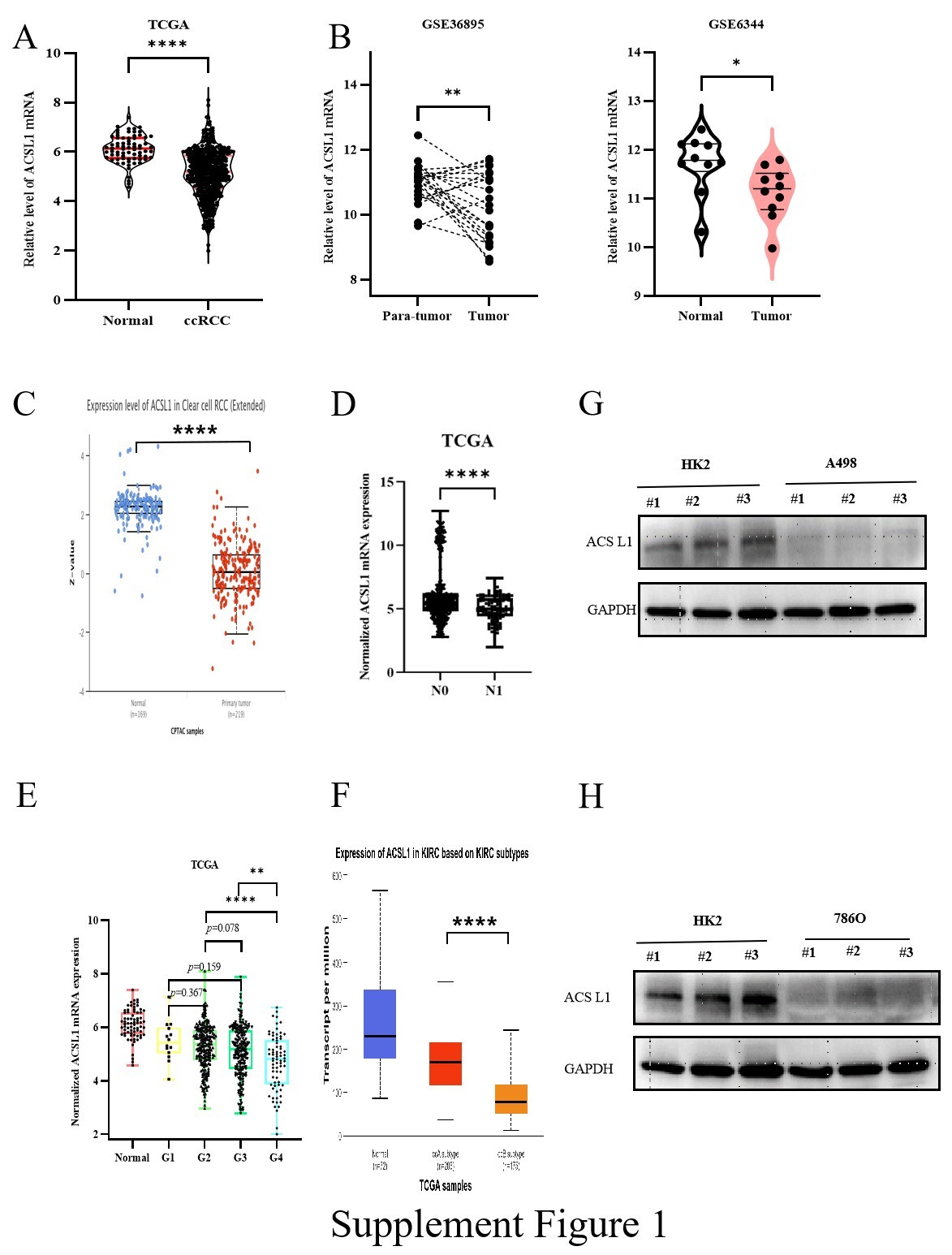

Supplement: Supplementary material — Supplementary Figure 1. Bioinformatic analysis revealed significant downregulation of ACSL1 expression in ccRCC. (A) Comparison of ACSL1 mRNA levels between ccRCC and adjacent normal kidney tissues in the TCGA dataset. (B) ACSL1 mRNA expression analysis in ccRCC versus normal kidney tissues from GSE36895 and GSE6344 datasets. (C) Differential ACSL1 protein expression between ccRCC and normal kidney tissues in the CPTAC database. (D) ACSL1 mRNA levels in lymph node metastatic (N1) versus non-metastatic (N0) ccRCC samples from the TCGA dataset. (E) Association between ACSL1 mRNA expression and pathological grades in the TCGA ccRCC cohort. (F) ACSL1 mRNA expression across molecular subtypes in the TCGA dataset, with the ccB subtype showing poorer prognosis. (G) Differential ACSL1 protein expression between HK2 and A498 cells. (H) Differential ACSL1 protein expression between HK2 and 786O cells. *P < 0.05, **P < 0.01, ***P < 0.001, ****P < 0.0001. [file KCBT_A_2567815_SM9536.jpg]

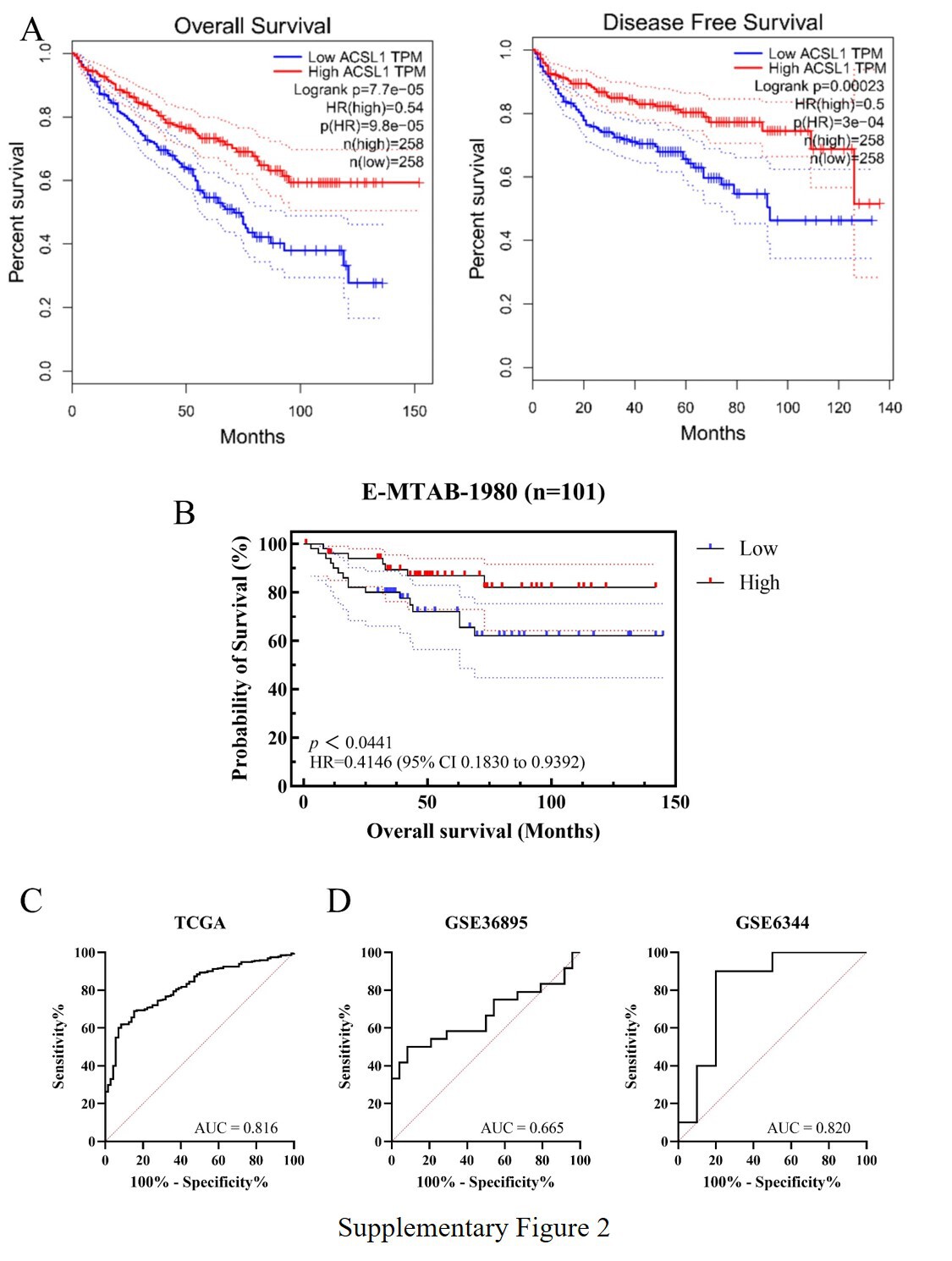

Supplement: Supplementary material — Supplementary Figure 2. ACSL1 expression is correlated with prognosis in ccRCC patients. (A) Kaplan‒Meier curves showing the OS and DFS of ccRCC patients stratified by ACSL1 expression levels in the TCGA dataset. (B) OS analysis based on ACSL1 expression in the ccRCC cohort from the E-MTAB-1980 dataset. (C) ROC curve analysis evaluating the diagnostic performance of ACSL1 expression in distinguishing ccRCC tumor tissues from normal kidney tissues in the TCGA dataset. (D) Diagnostic efficacy of ACSL1 expression assessed by receiver operating characteristic (ROC) curve analysis in GSE36895 and GSE6344 datasets. [file KCBT_A_2567815_SM9537.jpg]

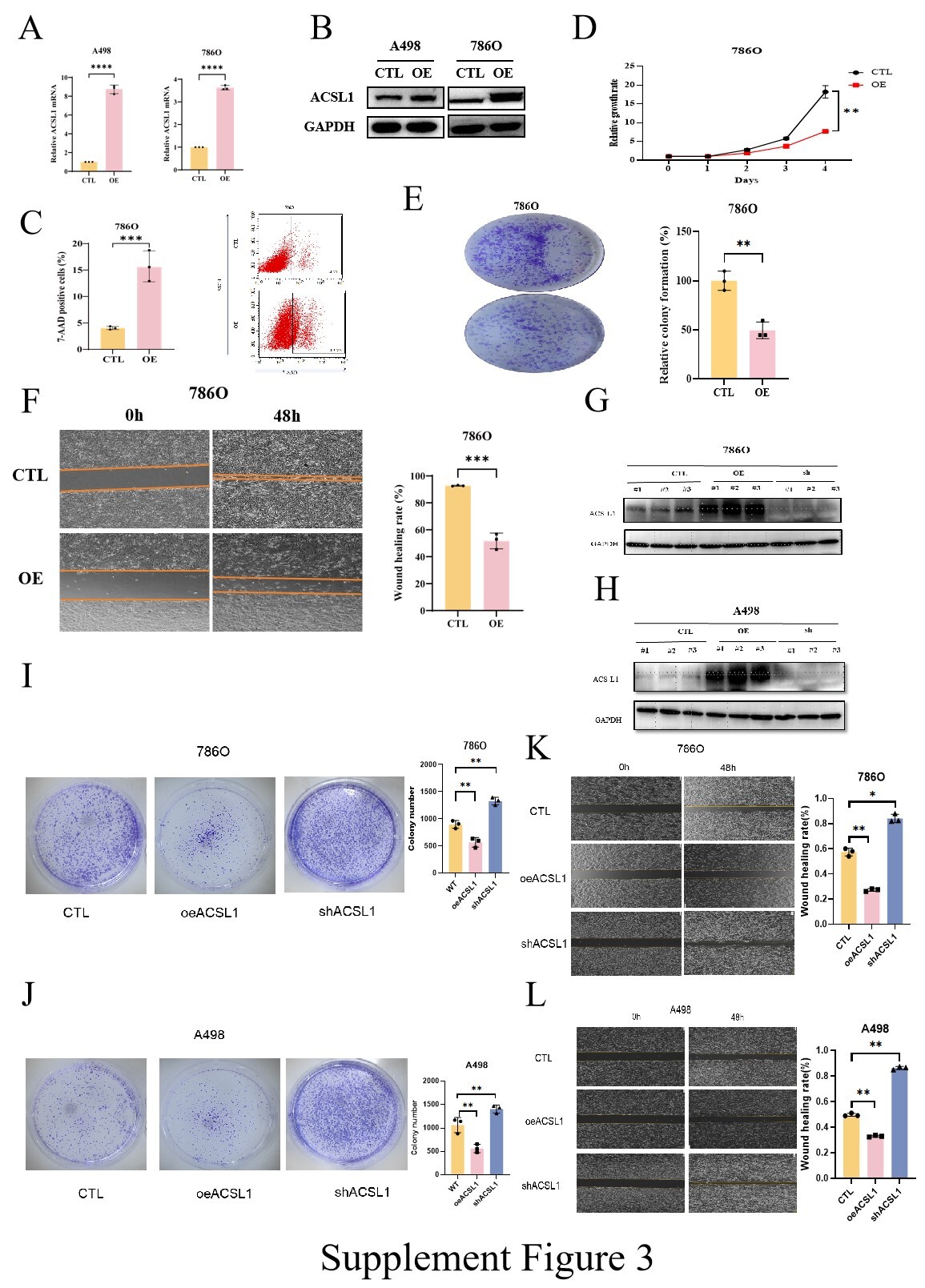

Supplement: Supplementary material — Supplementary Figure 3. ACSL1 suppresses tumor progression in ccRCC by inhibiting proliferation and migration. (A) ACSL1 mRNA expression was validated via qPCR in A498 and 786O cell lines with ACSL1 overexpression and was normalized to that of GAPDH. (B) Western blot analysis of ACSL1 protein levels in ACSL1-overexpressing A498 and 786O cells. (C) Flow cytometry analysis (right) and quantification (left) of 7-AAD-positive cells in 786O cells. (D) Cell proliferation was assessed by a CCK-8 assay in 786O cells. (E) Representative images (left) and quantitative analysis (right) of colony formation in 786O cells. (F) Scratch wound healing assay images (left) and migration rate quantification (right) in 786O cells at 48 h. (G) The overexpression and knockdown of ACSL1 were verified by Western blotting in 786O cells. (H) The overexpression and knockdown of ACSL1 were verified by Western blotting in A498 cells. (I) Representative images (left) and quantitative analysis (right) of colony formation in 786O cells. (J) Representative images (left) and quantitative analysis (right) of colony formation in A498 cells. (K) Scratch wound healing assay images (left) and migration rate quantification (right) in 786O cells at 48 h. (L) Scratch wound healing assay images (left) and migration rate quantification (right) in A498 cells at 48 h. *P < 0.05, **P < 0.01, ***P < 0.001, ****P < 0.0001. [file KCBT_A_2567815_SM9534.jpg]

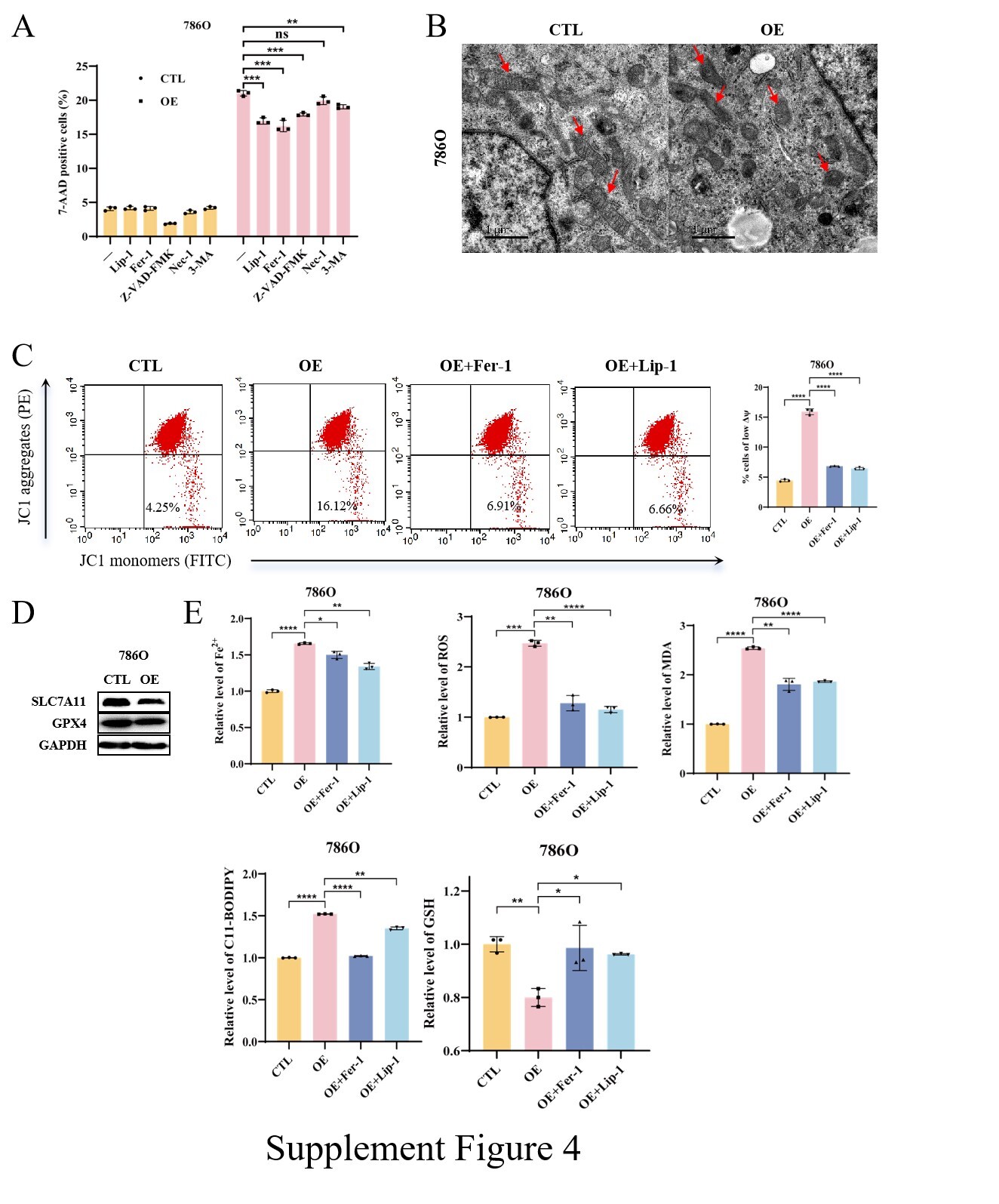

Supplement: Supplementary material — Supplementary Figure 4. Role of ACSL1 in promoting ferroptosis in ccRCC cells. (A) Flow cytometric analysis of 7-AAD-positive cell proportions in 786O cells treated with ferroptosis inhibitors (500 nM Lip-1 or 10 μM Fer-1), an apoptosis inhibitor (10 μM Z-VAD-FMK), a necroptosis inhibitor (10 μM Nec-1), or an autophagy inhibitor (2 mM 3-MA). (B) Representative transmission electron microscopy images of 786O cells (magnification = 11,500×; scale bar = 1 μm). Red arrowheads indicate mitochondria. (C) Quantitative analysis of JC-1 fluorescence intensity in 786O cells across treatment groups by flow cytometry. (D) Western blot analysis of ferroptosis-related proteins (SLC7A11 and GPX4) in ACSL1-overexpressing 786O cells. (E) Measurements of Fe²⁺, ROS, MDA, C11-BODIPY, and GSH levels in 786O cells under the indicated conditions. *P < 0.05, **P < 0.01, ***P < 0.001, ****P < 0.0001. [file KCBT_A_2567815_SM9533.jpg]

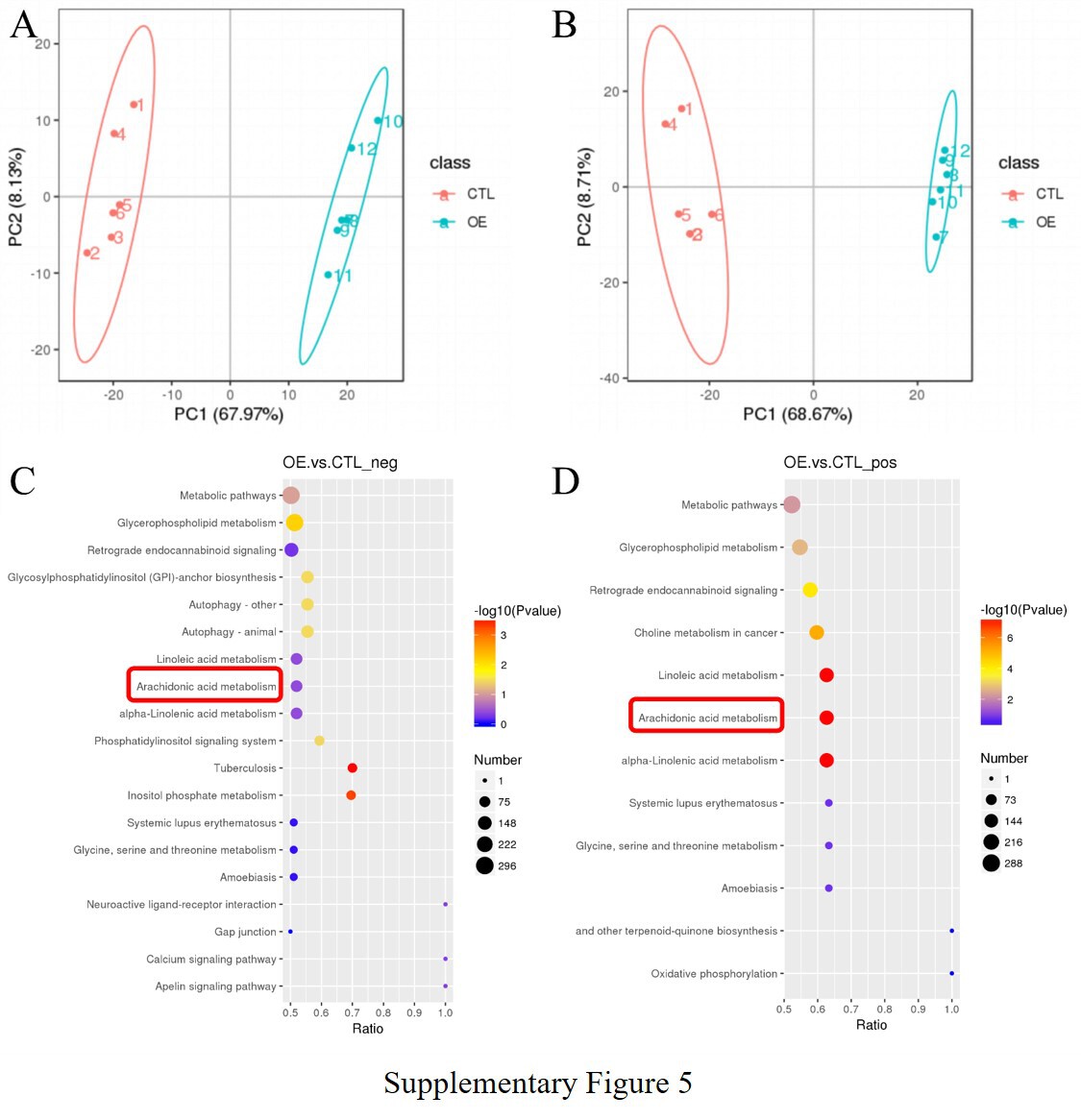

Supplement: Supplementary material — Supplementary Figure 5. Untargeted lipidomics analysis revealed ACSL1-induced enhancement of ferroptosis-related lipid metabolism. (A, B) Principal component analysis (PCA) plots of untargeted lipidomics data in negative ion mode (A) and positive ion mode (B) (n = 6 per group). (C, D) Pathway enrichment analysis of differentially abundant lipids between the ACSL1-overexpressing (OE) and control (CTL) groups in negative ion mode (C) and positive ion mode (D). [file KCBT_A_2567815_SM9539.jpg]

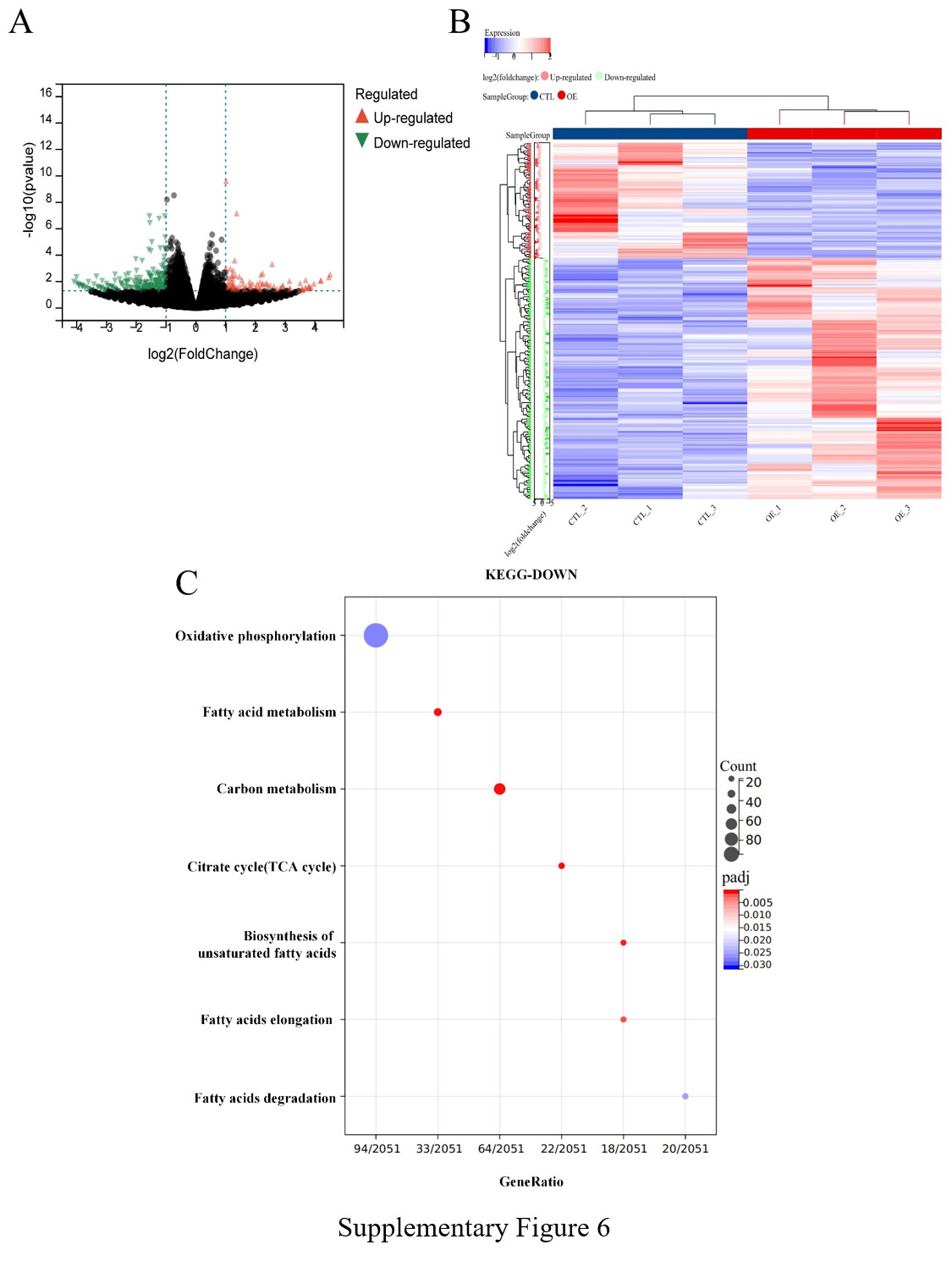

Supplement: Supplementary material — Supplementary Figure 6. Transcriptomics analysis suggested that ACSL1 exerts tumor-suppressive effects via the p53 pathway. (A) Volcano plot and (B) heatmap of differentially expressed genes identified by transcriptomic sequencing (n = 3 per group). (C) Bubble plot of KEGG pathway enrichment analysis for downregulated genes. [file KCBT_A_2567815_SM9535.jpg]

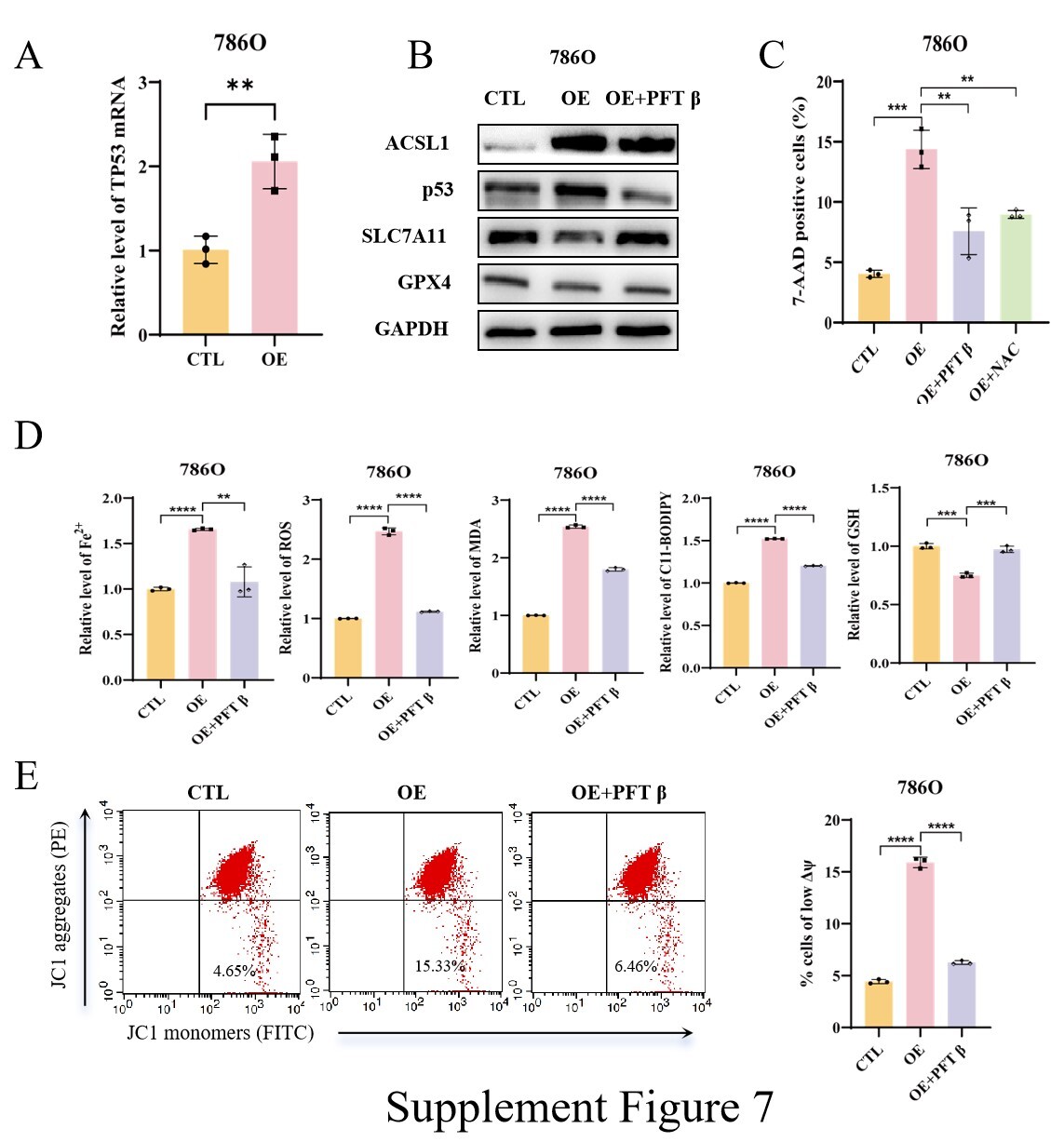

Supplement: Supplementary material — Supplementary Figure 7. ACSL1 exerts its tumor-suppressive role by activating p53. (A) TP53 mRNA expression levels in 786O cells after ACSL1 overexpression. (B) Western blot analysis of SLC7A11 and GPX4 protein levels in 786O cells treated with the p53-specific inhibitor PFTβ (10 μM). (C) Flow cytometry quantification of 7-AAD-positive cells in 786O cells treated with PFTβ and the ROS scavenger NAC (5 mM). (D) Measurements of intracellular Fe²⁺, ROS, MDA, C11-BODIPY, and GSH levels in PFTβ-treated 786O cells. (E) Flow cytometry analysis of JC-1 staining (mitochondrial membrane potential) and corresponding statistical results in PFTβ-treated 786O cells. *P < 0.05, **P < 0.01, ***P < 0.001, ****P < 0.0001. [file KCBT_A_2567815_SM9540.jpg]

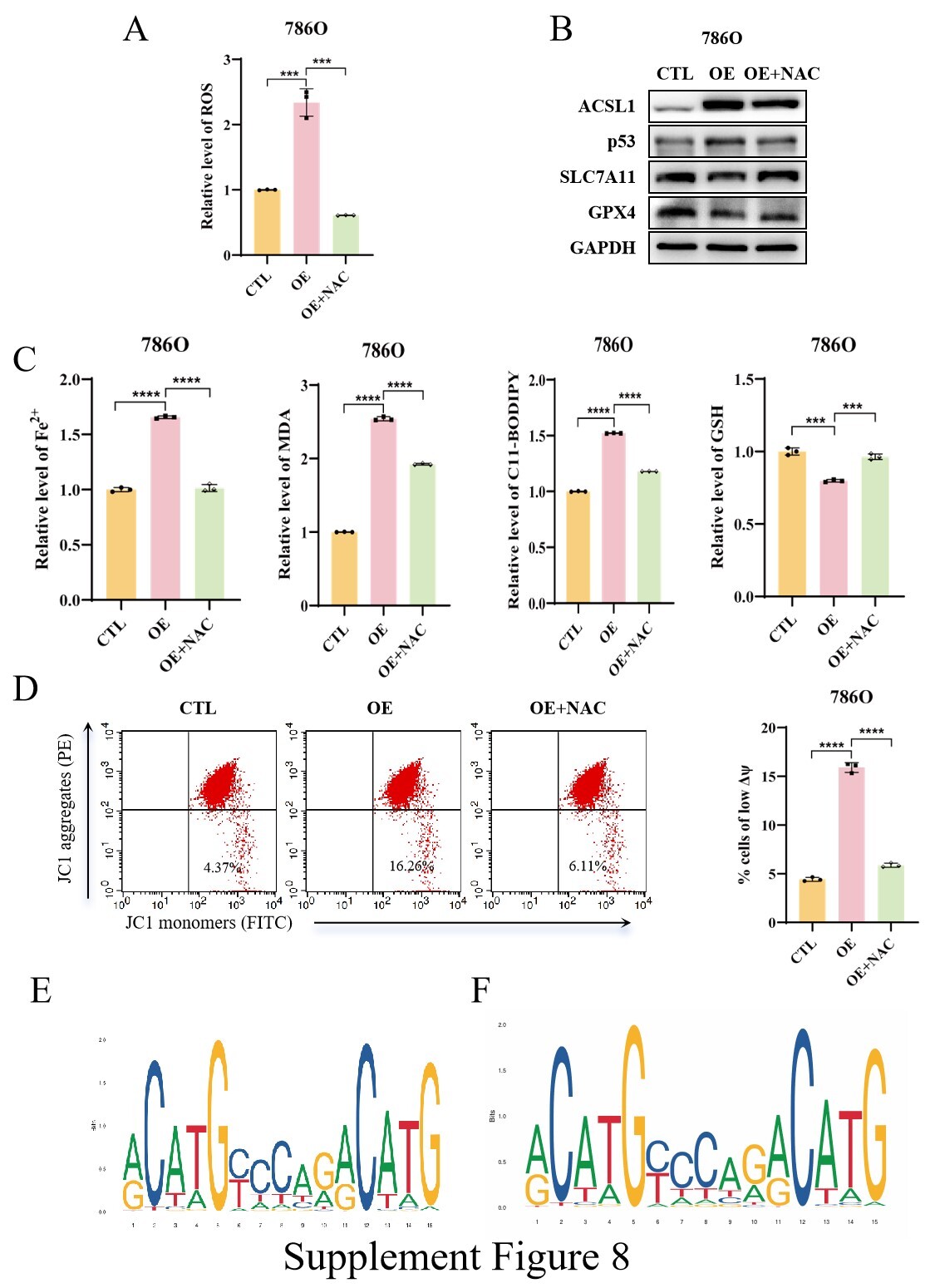

Supplement: Supplementary material — Supplementary Figure 8. Attenuation of ACSL1-induced ferroptosis by reducing intracellular ROS. (A) Flow cytometry quantification of ROS levels in 786O cells treated with the ROS scavenger NAC (5 mM). (B) Representative Western blot images showing the protein expression levels of p53, SLC7A11, and GPX4 in NAC-treated 786O cells. (C) Measurements of intracellular Fe²⁺, MDA, C11-BODIPY, and GSH levels in NAC-treated 786O cells. (D) Flow cytometry analysis of JC-1 staining (mitochondrial membrane potential) in PFTβ-treated 786O cells. (E) Bioinformatic analysis predicted potential p53-binding sites within the SLC7A11 promoter region. (F) Bioinformatic analysis predicted potential p53-binding sites within the GPX4 promoter region. *P < 0.05, **P < 0.01, ***P < 0.001, ****P < 0.0001. [file KCBT_A_2567815_SM9541.jpg]
