# Supplementary material for: Loss of ACSL1 fuels ferroptosis resistance in clear cell renal carcinoma
Source: Cancer Biol Ther. 2025 Oct 6;26(1):2567815. doi: 10.1080/15384047.2025.2567815 (PMC12505508; doi:10.1080/15384047.2025.2567815)
Supplement: Supplementary material — s CLEAN COPY. [file KCBT_A_2567815_SM9538.docx]

Supplemental Materials and Methods

**Loss of ACSL1 fuels ferroptosis resistance in clear cell renal carcinoma**

Western blot analysis, immunohistochemistry, and immunofluorescence

Cell lysates were prepared using RIPA buffer (Solarbio, China, R0020) supplemented with 1% protease inhibitor mixture (Solarbio, China, P6730) and 1% phosphatase inhibitor (Solarbio, China, P1260). Proteins were separated on 5–15% gradient gels, transferred to hydrophobic PVDF transfer membranes (Merck, Germany, ISEQ00010), and probed with antibodies detailed in Supplementary Table 1. HRP-conjugated Affinipure Goat Anti-Rabbit IgG (Proteintech, China, SA00001-2) or HRP-conjugated Affinipure Goat Anti-Mouse IgG (Proteintech, China, SA00001-1) served as secondary antibodies. Blots were visualized using enhanced chemiluminescence (Solarbio, China, ECL Western Blotting Substrate, PE0010). The ECL imaging equipment includes the Tanon 4600 (shanghai, China) and the GeneGnome HR model No.7000 (Synoptics Ltd, UK).

Collected tissues were fixed in formalin for 1 week and paraffin-embedded sections were prepared for subsequent histologic examination.

The initial steps for both immunohistochemical staining and immunofluorescence staining were identical. Paraffin sections were first dewaxed and hydrated. Antigens were repaired in 10 mM sodium citrate solution at high temperature (95°C) for 5 minutes and then cooled to room temperature.

For immunohistochemical staining, endogenous peroxidase activity was quenched using 3% H_2_O_2_ for 15 minutes. Subsequently, sections were blocked with 0.2% fish skin gelatin for 30 minutes at room temperature. They were then incubated overnight at 4°C with primary antibodies (details regarding specific antibody types and dilution concentrations are provided in Supplementary Table 1). The following day, after a 15-minute incubation with secondary antibodies at room temperature, streptavidin-peroxidase solution was applied for an additional 15 minutes. Finally, staining was visualized using either 3,3'-Diaminobenzidine (DAB, Solarbio, DA1016) or 3-amino-9-ethylcarbozole (AEC, Solarbio, A2010). Final hematoxylin staining, differentiation and bluing were used to label the nucleus. It is noteworthy that all the mentioned reagents were sourced from the SP kits (Broad Spectrum) provided by Solepol (SP0041).

In the immunofluorescence staining, sections were initially blocked with 5% normal goat serum for 30 minutes at room temperature. Subsequently, sections were incubated overnight at 4°C with primary antibodies (specific antibody types and dilution concentrations are provided in Supplementary Table 1). On the following day, sections were washed with PBS and incubated with fluorescent secondary antibodies (see Supplementary Table 1 for details). Additionally, nuclei were stained with DAPI, and proximal tubules were stained with fluorescein lotus tetragonolobus lectin (LTL, 1:200, FL1321, Vector Laboratories) for 60 minutes.

Supplementary Table 1 Detailed information on antibodies and other dyes

| Antibodies | Company | product number | Dilution ratio of western blot | Dilution ratio of immunohistochemistry | Dilution ratio of immunofluorescence |
| --- | --- | --- | --- | --- | --- |
| ACSL1 | Proteintech, China | 13989-1-AP | 1:1000 | 1:250 | 1:250 |
| GAPDH (Rabbit) | Proteintech, China | 10494-1-AP | 1:2000 | - | - |
| β-Actin (Rabbit) | Proteintech, China | 81115-1-RR | 1:2000 | - | - |
| p53 | Santa Cruz Biotechnology | C1623 | 1:5000 | - | - |
| SLC7A11 | Proteintech, China | 26864-1-AP | 1:1000 | - | - |
| GPX4 | Abmart | T56959F | 1:2000 | - | - |
| DAPI | Solarbio, China | C0060 | - | - | 1:1000 |
| CoraLite594 – conjugated Goat Anti-Rabbit IgG | Proteintech, China | SA00013-4 | - | - | 1:200 |

Real-time quantitative polymerase chain reaction analysis

We extracted total RNA using the Total RNA Extractor kit (Solarbio, China, R1200). Subsequently, 1 μg of total RNA was reverse-transcribed using the cDNA Synthesis SuperMix Kit (YEASEN, China, 11137ES60). Real-time quantitative polymerase chain reaction (RT-qPCR) analysis was conducted using the Hieff® System (YEASEN, China, 11203ES08) with SYBR Green Master Mix (High Rox Plus), following 3-step standard cycling conditions and employing sequence-specific primers listed in Supplementary Table 2. Melting curve analysis was performed to confirm the amplification of a single product. For quantitative analysis, all samples were normalized to GAPDH gene expression using the ΔΔCT method.

Supplementary Table 2. Primer sequence details table

| Gene | Forward primerz | Reverse primer |
| --- | --- | --- |
| ACSL1 (Homo sapiens) | GGAAGAGCCAACAGACGGAA | TTTAGCTCCATGACACAGCA |
| TP53 (Homo sapiens) | TGGCCATCTACAAGCAGTCA | GGTACAGTCAGAGCCAACCT |
| GAPDH (Homo sapiens) | GGAAGGTGAAGGTCGGAGTC | TGGAATTTGCCATGGGTGGA |

Pharmacological compounds and test kits

The information of various test kits and compounds used in this study is as follows.

Supplementary Table 3. Main reagents and consumables involved in the experiment

| **Item** | **Product number** | **Company** |
| --- | --- | --- |
| ROS fluorescence probe-DHE | R001 | Vigorous Biotechnology Beijing Co., Ltd. |
| C11 BODIPY 581/591, Cell lipid peroxidation probe | FMK12930 | BIOFOUNT |
| 7-AAD Cell viability test kit | C1053S | Beyotime Biotechnology Co., Ltd. |
| MDA kit | BC0020 | Beijing Suolaibao Technology Co., Ltd. |
| Fe^2+^ kit | CA1530 | Beijing Suolaibao Technology Co., Ltd. |
| Reduced glutathione (GSH) test kit | BC1175 | Beijing Suolaibao Technology Co., Ltd. |
| Enhanced Mitochondrial Membrane Potential Test Kit (JC-1) | C2003S | Beyotime Biotechnology Co., Ltd. |
| Pifithrin-β hydrobromide（PFTβ） | HY-16702A | MedChemExpress Co., Ltd. |
| Ferrostatin-1（Fer-1） | HY-100579 | MedChemExpress Co., Ltd. |
| Liproxstatin-1（Lip-1） | HY-12726A | MedChemExpress Co., Ltd. |
| Z-VAD-FMK | HY-16658B | MedChemExpress Co., Ltd. |
| Necrostatin-1（Nec-1） | HY-15760 | MedChemExpress Co., Ltd. |
| 3-Methyladenine（3-MA） | HY-19312 | MedChemExpress Co., Ltd. |
| Acetylcysteine（NAC） | HY-B0215 | MedChemExpress Co., Ltd. |
